# PET imaging of focused-ultrasound enhanced delivery of AAVs into the murine brain

**DOI:** 10.7150/thno.85549

**Published:** 2023-09-25

**Authors:** Javier Ajenjo, Jai Woong Seo, Josquin Foiret, Bo Wu, Marina Nura Raie, James Wang, Brett Zain Fite, Nisi Zhang, Rim Malek, Corinne Beinat, Noeen Malik, David Alexander Anders, Katherine W. Ferrara

**Affiliations:** 1 Molecular Imaging Program at Stanford (MIPS), Department of Radiology, School of Medicine, Stanford University, Stanford, CA, USA.; 2 Stanford Cyclotron & Radiochemistry Facility (CRF), Department of Radiology, School of Medicine, Stanford University, Stanford, CA, USA.

**Keywords:** Gene therapy, Adeno-associated virus, Focused ultrasound, Blood-brain barrier, Positron emission tomography

## Abstract

**Rationale:** Despite recent advances in the use of adeno-associated viruses (AAVs) as potential vehicles for genetic intervention of central and peripheral nervous system-associated disorders, gene therapy for the treatment of neuropathology in adults has not been approved to date. The currently FDA-approved AAV-vector based gene therapies rely on naturally occurring serotypes, such as AAV2 or AAV9, which display limited or no transport across the blood-brain barrier (BBB) if systemically administered. Recently developed engineered AAV variants have shown broad brain transduction and reduced off-target liver toxicity in non-human primates (NHPs). However, these vectors lack spatial selectivity for targeted gene delivery, a potentially critical limitation for delivering therapeutic doses in defined areas of the brain. The use of microbubbles, in conjunction with focused ultrasound (FUS), can enhance regional brain AAV transduction, but methods to assess transduction in vivo are needed.

**Methods:** In a murine model, we combined positron emission tomography (PET) and optical imaging of reporter gene payloads to non-invasively assess the spatial distribution and transduction efficiency of systemically administered AAV9 after FUS and microbubble treatment. Capsid and reporter probe accumulation are reported as percent injected dose per cubic centimeter (%ID/cc) for *in vivo* PET quantification, whereas results for *ex vivo* assays are reported as percent injected dose per gram (%ID/g).

**Results:** In a study spanning accumulation and transduction, mean AAV9 accumulation within the brain was 0.29 %ID/cc without FUS, whereas in the insonified region of interest of FUS-treated mice, the spatial mean and maximum reached ~2.3 %ID/cc and 4.3 %ID/cc, respectively. Transgene expression assessed *in vivo* by PET reporter gene imaging employing the pyruvate kinase M2 (PKM2)/[^18^F]DASA-10 reporter system increased up to 10-fold in the FUS-treated regions, as compared to mice receiving AAVs without FUS. Systemic injection of AAV9 packaging the EF1A-PKM2 transgene followed by FUS in one hemisphere resulted in 1) an average 102-fold increase in PKM2 mRNA concentration compared to mice treated with AAVs only and 2) a 12.5-fold increase in the insonified compared to the contralateral hemisphere of FUS-treated mice.

**Conclusion:** Combining microbubbles with US-guided treatment facilitated a multi-hour BBB disruption and stable AAV transduction in targeted areas of the murine brain. This unique platform has the potential to provide insight and aid in the translation of AAV-based therapies for the treatment of neuropathologies.

## Introduction

Within the field of cell and gene therapy, the use of recombinant adeno-associated viruses (AAVs) has emerged as the most promising therapeutic approach for a wide range of neurological disorders. AAVs contain single-stranded deoxyribonucleic acid (ssDNA) within ~25 nm protein capsids and have a cargo capacity of ~4.7 kb. AAV vector-based genetic intervention has shown remarkable success for the clinical treatment of retinal dystrophy (Luxturna^®^, Roche), spinal muscular atrophy (Zolgensma^®^, Novartis AG) in children and recently, hemophilia B (Hemgenix^®^, CSL Behring LLC) 1-4. Despite more than 250 clinical trials ongoing worldwide, AAV-based gene therapy for the treatment of neurological disorders in adults, however, has not yet been approved 5. Genetic intervention employing naturally occurring AAV serotypes (i.e., AAV2, AAV9) aimed at the central nervous system (CNS) is hampered by their limited transport across the protective blood-brain barrier (BBB) when systemically administered. Traditional methods for delivery of AAVs, such as intracranial injections, are invasive, have limited precision, and systemic delivery can result in off-target effects and toxicity. Recently, novel-engineered AAV variants, such as PHP.eB and CAP.B10, which are evolved from AAV9, have shown efficient and widespread neuronal transduction across the CNS 6-8. However, these re-engineered vectors are not regionally specific in transgene delivery.

Focused ultrasound (FUS) with injected microbubbles (MBs) can transiently disrupt the BBB with potential to deliver AAVs into the brain 9-17. As a result of MB insonation, BBB permeability is increased by mechanisms that include mechanical stress on cell membranes and stretching of tight junctions between endothelial cells, enhancing transport 18. Historically, uptake and protein expression of AAVs locally delivered into the brain using FUS have been assessed using invasive techniques 12. In our previous work, AAVs radiolabeled with trace amounts of a positron emitter enabled non-invasive tracking of AAV capsids through *in vivo* imaging with positron emission tomography (PET) 19 and further allowed us to evaluate the receptor for AAV9 through tracking of AAVs in transgenic mice 20. AAV capsids with a PET imaging tag and reporter gene facilitated a non-invasive comparison of localization and transduction in the mouse brain and liver 21. Furthermore, PET reporter imaging allowed us to monitor and confirm transduction and protein expression *in vivo* over more than one year.

In this work, we investigate optimal FUS parameters for efficient and targeted AAV delivery into local areas of the murine brain after systemic administration of AAV9, a naturally-occurring AAV serotype with limited transport across the BBB 22. PET methods allow us to optimize and quantify capsid accumulation and protein expression in sonicated areas of the brain. This unique imaging platform allows us to spatially map both capsid accumulation and transduction efficiency *in vivo* in treated and contralateral areas of the brain. We apply pyruvate kinase M2 (PKM2) as a PET reporter gene 23, 24. PKM2 catalyzes the conversion of phosphoenolpyruvate to pyruvate in the last step of glycolysis and has low expression in the healthy brain. In our previous work, we imaged PKM2 by PET using 1-((2-fluoro-6-[^18^F]fluorophenyl)sulfonyl)-4-((4-methoxyphenyl)sulfonyl)piperazine ([^18^F]DASA-23), which can freely cross the BBB and has recently undergone a phase 1 clinical trial for patients with intracranial tumors (NCT03539731) and in this study we apply a related DASA analog (1-((2-fluoro-6-[^18^F]fluorophenyl)sulfonyl)-4-((2,3-dihydrobenzo[*b*][1,4]dioxin-6-yl)sulfonyl)piperazine, [^18^F]DASA-10) with superior pharmacokinetics 25. Herein, we deliver the EF1A-PKM2 transgene within the AAV9 capsid while promoting transport with FUS-enabled BBB opening. To assess the FUS-enhanced delivery, we used our PET imaging platform to measure reporter protein expression. Furthermore, we evaluate the spatial transduction map of PKM2 and mNeonGreen (mNG) with fluorescent imaging and compared with the PET imaging data.

Assessing local AAV uptake and transgene protein synthesis from mRNA *in vivo* is crucial for preclinical and clinical evaluation of targeted gene therapy, as it can provide insight for injected dose and the number of genome copies delivered. Tailoring of target gene delivery to the needs of particular diseases and patient populations is essential to minimize potential adverse effects while still achieving a therapeutic benefit. Toxicity has been dose limiting for AAVs and therefore dose reduction is desirable and could potentially be accomplished through FUS-enhanced accumulation 26.

## Results

### Passive acoustic mapping confirms BBB opening in sonicated area

**Figure 1:**
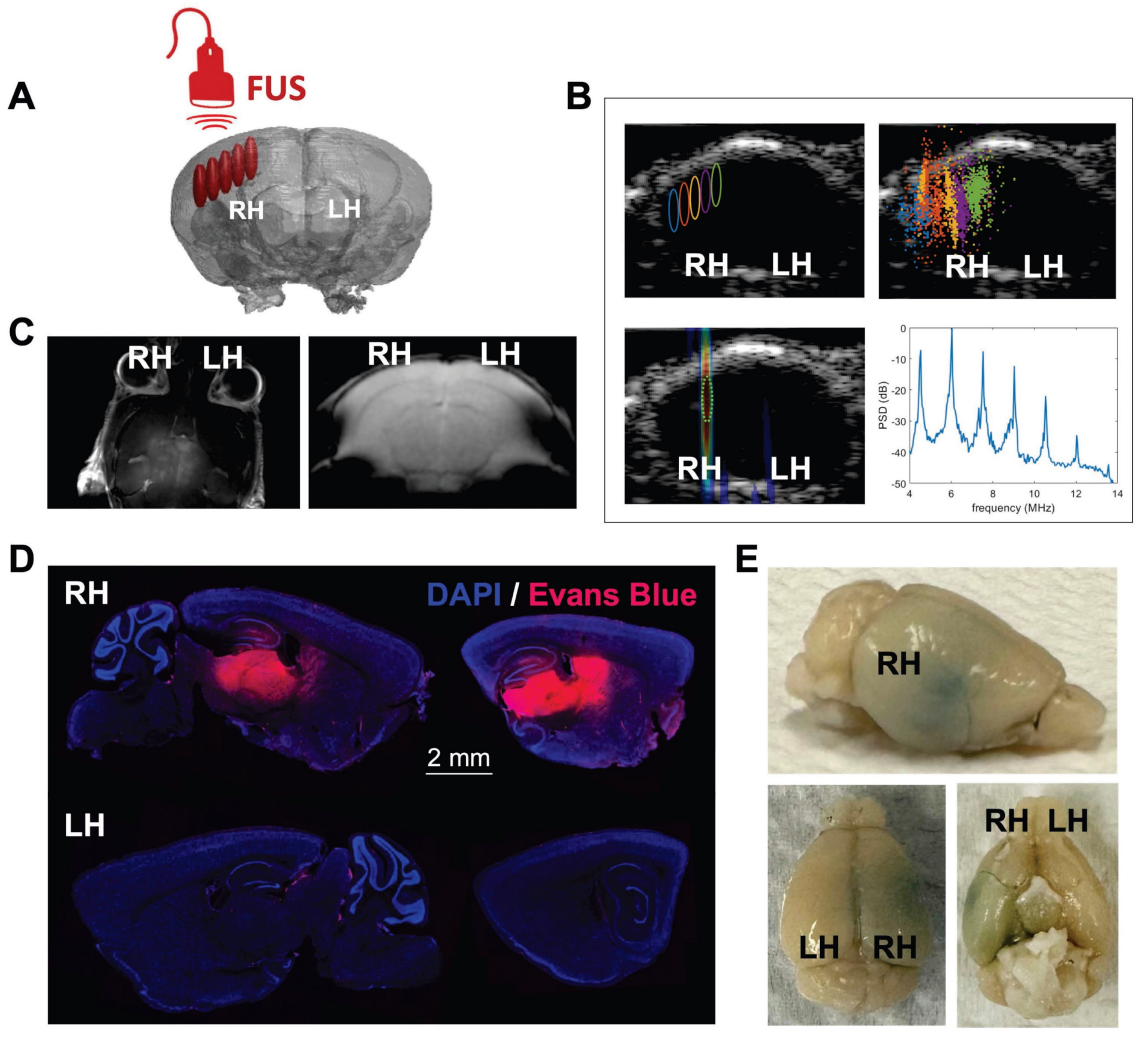
** Focused ultrasound treatment and BBB opening study by optical imaging. A.** Illustration of FUS target area in the right hemisphere of the mouse brain. The beam follows a dorsal trajectory. **B.** Pre-treatment ultrasound-guided FUS target area (upper-left panel), post-FUS map localizing acoustic emissions from MBs for the entire treatment (upper-right panel), real-time passive acoustic monitoring (PAM) (lower-left panel) and power spectral density (PSD) plot localizing acoustic emissions from MBs for the entire treatment (lower-right panel) during FUS treatment. **C.** MRI 30 minutes post-FUS treatment (600 kPa) employing gadoteridol as contrast agent (left image), T2*-weighted MRI 30 minutes post-FUS treatment (600 kPa) (right image). **D.** Fluorescence microscopy (DAPI/Evans Blue) images of brain slices (100 μm) showing the FUS-treated (right) and contralateral (left) hemispheres. **E.** Brain images after FUS treatment followed by Evans Blue dye injection. **Abbreviations**: LH: left hemisphere. RH: right hemisphere.

As an overview of the visualization and guidance methods, we applied ultrasound-guided focused-ultrasound (USg-FUS) and microbubble (MB) treatment to target the hippocampal region in the brain of C57BL/6 mice, following injection of Evans Blue (EB) dye 27 as contrast agent (Fig. 1). The FUS-target area was determined employing B-mode imaging based on the anatomical features of the animal (Supplementary Fig. S1A), in the supine position and contrast pulse sequencing (CPS) to monitor MB circulation, and radiofrequency signals were passively recorded during FUS treatment with the imaging transducer (Fig. 1A). Ultrasound guidance and passive acoustic mapping (PAM) during sonication were performed with a L12-5 transducer (38 mm aperture, Phillips/ATL) positioned in the central opening of the 2D therapeutic array (Supplementary Fig. S1B), the mechanical index remaining equal to or less than 0.11 during imaging. A unique 128-element 2D therapeutic array with a 1.5 MHz center frequency then directed the pulse train to the region of interest (ROI). The FUS treatment consisted of sweeping the focus across a square grid of 5 x 5 positions with steps of 0.5 mm (insonified volume ~2.5 x 2.5 x 2.7 mm^3^ = 17 mm^3^) with a 1 ms burst at a given pressure for each spatial position (i.e., 25 ms to cover the 25-point grid) with the grid tilted to follow the curvature of the skull. The grid repetition rate was 5 Hz and the total sonication time was 2 min, leading to a total of 600 1-ms bursts for each grid point.

Localization of cavitation events by PAM was conducted employing the angular spectrum approach (ASPAM) 28 providing a spatial assessment of MB insonation (Fig. 1B) and a spectral view of the presence of discrete harmonics. Such harmonics verify that the insonation results in stable, rather than inertial, cavitation. T2*-weighted magnetic resonance imaging (MRI) was incorporated to verify that insonation did not result in red cell extravasation (Fig. 1C). Sonicated areas spatially coincided with increased accumulation of EB dye (Fig. 1D-E) in the targeted area of the treated hemisphere after brain collection and fluorescence microscopy image analysis.

### FUS optimization with [^64^Cu]Cu-AAV9

**Figure 2:**
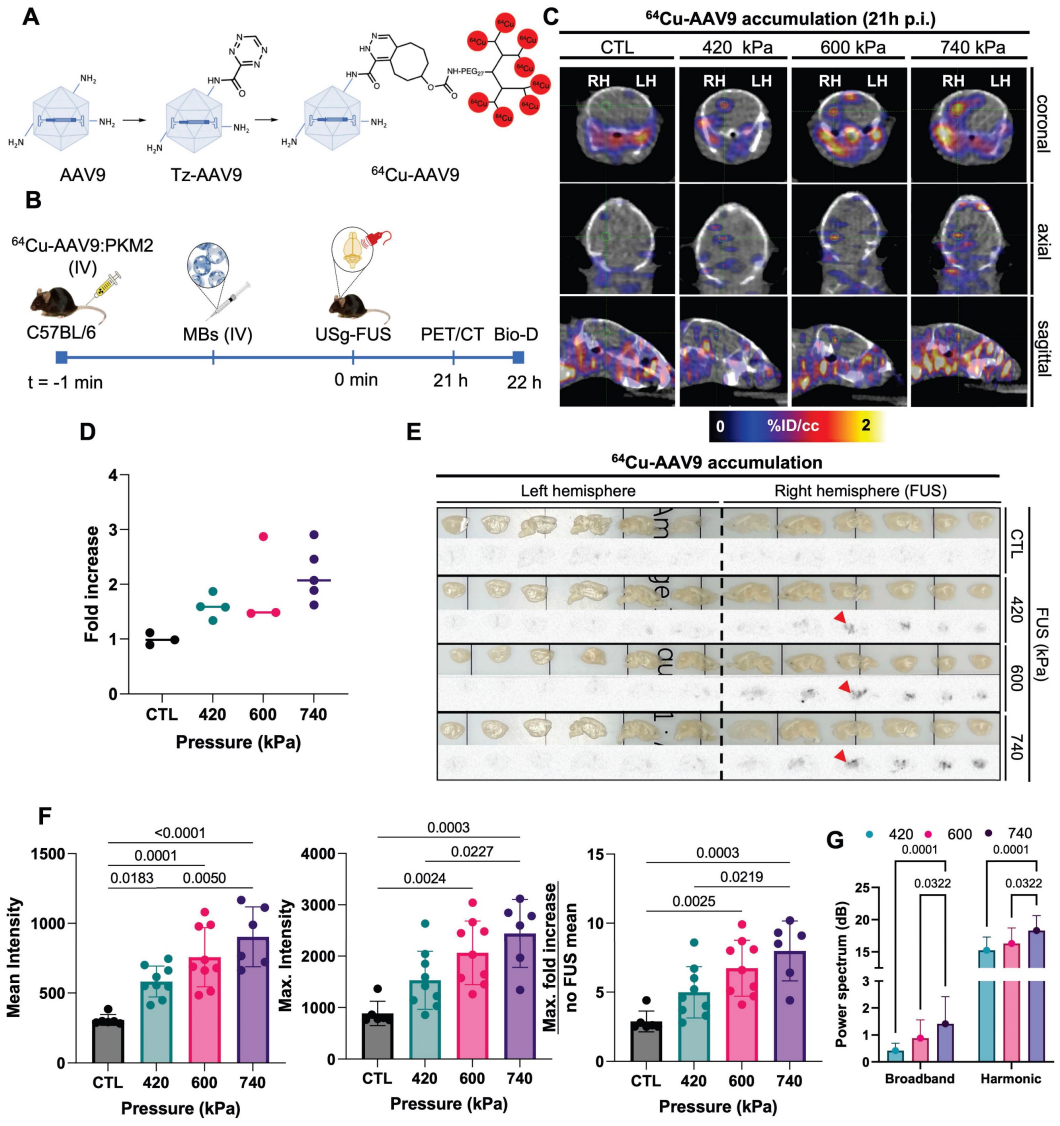
**Optimization of FUS treatment parameters and *ex vivo*/*in vivo* AAV accumulation study. A.** Radiolabeling scheme of AAV9 by modification of lysine residues in the surface of the capsid with a peptide-multichelator via “click chemistry”. **B.** Schematic illustration of radiolabeled-AAV brain delivery and accumulation study. ^64^Cu-AAV9 is systemically injected in C57BL/6 mice (n_CTL_ = 3, n_420_ = 4, n_600_ = 3, n_740_ = 5) and capsid delivery and accumulation were assessed by PET/CT imaging (*in vivo*) and biodistribution (Bio-D)/autoradiography analysis (*ex vivo*). **C.** Representative coronal, sagittal and axial PET/CT images at 21 h post injection (p.i.) of ^64^Cu-AAV9 of FUS-treated (420 kPa, 600 kPa, 740 kPa) and no-FUS AAV9-injected control (CTL) mice. **D.** Fold increase in ^64^Cu-AAV9 accumulation within the entire FUS-treated brain hemisphere (RH). **E.** *Ex vivo* autoradiography at 22 h p.i. of ^64^Cu-AAV9 at different FUS pressures and without FUS treatment (CTL). Red arrowheads highlight the insonified region in the central slice. **F.** Intensity and fold increase as assessed from autoradiography. **G**. Power spectra as a function of the treatment time for the different groups of pressure and all observations in all animals (420, 600, 740 kPa) **Abbreviations**: FUS: Focused ultrasound. HPC: hippocampus. LH: left hemisphere. RH: right hemisphere. IV: intravenous injection. MBs: microbubbles, CTL: Control. F. Ordinary one-way ANOVA and G. Two-way ANOVA each with multiple comparison tests. *p*-values are presented in the figures.

Radiolabeling of AAV9 was accomplished by employing an in-house developed peptide-based multichelator approach as in 21 (Fig. 2A). First, the exposed lysine residues on the AAV9 surface (420-480 residues/viral particle) were modified by conjugation with a tetrazine-NHS ester (300 equivalents) followed by purification by dialysis. The resulting tetrazine-AAV (Tz-AAV) was attached to a TCO-multichelator (8 equivalents) by biorthogonal conjugation. After purification by a centrifugal filter, the labeling reaction afforded [^64^Cu]Cu-(NOTA)_8_-AAV9 with an overall 51% yield. For simplicity, [^64^Cu]Cu-(NOTA)_8_-AAV9 will be denoted as ^64^Cu-AAV9. The modification of the AAV9 surface as a conjugate platform did not alter the transduction efficiency of the capsid, as shown by *in vitro* assays in our previous work 19.

We then evaluated capsid localization by PET/CT imaging, biodistribution, and autoradiography with ^64^Cu-AAV9 (Fig. 2B) in C57BL/6 mice (n = 15). Based on image analysis at 21 h post injection (p.i.), radiolabeled capsids accumulated in sonicated areas (right brain hemisphere, RH) of mice treated with 420 kPa, 600 kPa and 740 kPa FUS pressures (Fig. 2C). The highest ^64^Cu-AAV9 accumulation was achieved at a FUS pressure of 740 kPa, while accumulation was not found in analogous areas of the no-FUS, AAV-injected control (CTL) mouse brains. On biodistribution at 22 h p.i., ^64^Cu-AAV9 uptake, quantified as percent injected dose per gram (%ID/g), was enhanced in the entire sonicated hemisphere of the brain by 1.5-, 1.9- and 2.2-fold for 420, 600 and 740 kPa FUS pressures, respectively (Fig. 2D). *Ex vivo* autoradiography confirmed increased radiolabeled capsid accumulation in the right hemisphere of the brain of all FUS-treated subjects, in contrast to the contralateral (left hemisphere, LH) region, correlating with the results obtained by PET imaging and biodistribution study analysis (Fig. 2E). The local enhancement in accumulation, as assessed by autoradiography, was 6.7- and 7.0-fold for 600 and 740 kPa, respectively (Fig. 2F). In contrast, relative uptake between treated and contralateral hemispheres remained similar for mice treated with AAVs without FUS. While ^64^Cu-AAV9 accumulation was greater when FUS was performed at 740 kPa, immunohistochemical analysis revealed red blood cell extravasation at this pressure (Supplementary Fig. S2). Additionally, T2*-weighted MRI 29 30 minutes after sonication and analysis of the hematoxylin and eosin-stained (H&E) sagittal brain slices of sonicated hemispheres confirmed the absence of red-cell extravasation and apoptosis with the 600 kPa pressure (Supplementary Fig. S2 and Fig. S3). We monitored the cavitation emissions as described in Supplementary Fig. S3-S5. The power spectral density confirmed an increase in the broadband spectral content with 740 kPa insonation as compared with either 410 or 600 kPa (Supplementary Fig. S6). Therefore, 600 kPa was used as the pressure of reference in subsequent experiments.

### Local brain accumulation of radiolabeled-AAV9 was enhanced in sonicated areas upon PET imaging of tagged capsids

**Figure 3:**
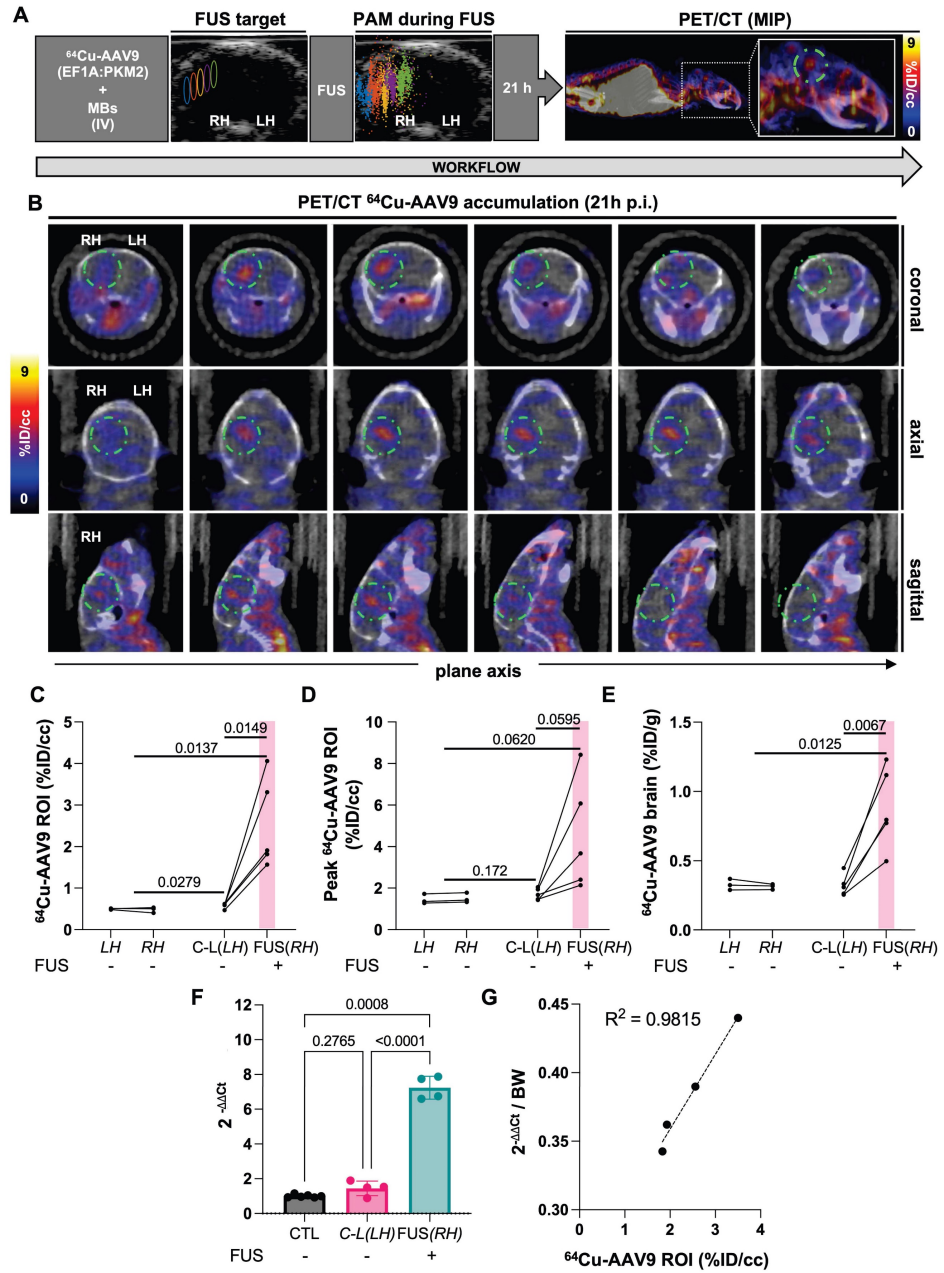
**^64^Cu-AAV9 accumulation study through *in vivo* quantitative PET/CT imaging and *ex vivo* biodistribution analysis. A.** USg-FUS target and post-FUS cavitation localization area from PAM, maximum intensity projection (MIP) PET/CT image at 21 h post ^64^Cu-AAV9 systemic injection in C57BL/6 mice. **B.** Representative coronal, axial and sagittal plane PET/CT images of an example mouse at 21 h post injection (p.i.) of ^64^Cu-AAV9 after brain lateral FUS treatment (600 kPa). Radiotagged AAV capsid accumulation has been highlighted with a dotted green circle for clarity. **C.** Image-based blood subtracted radioactivity (%ID/cc) in the brain region of interest (ROI, 10-11 mm^3^) of FUS-treated AAV9-injected (n = 5) and no-FUS AAV9-injected (n = 3) C57BL/6 mice at 21 h p.i. **D.** Image-based blood subtracted peak radioactivity (%ID/cc) in the brain ROI (10-11 mm^3^) of FUS-treated AAV9-injected (n = 5) and no-FUS AAV9-injected (n = 3) C57BL/6 mice at 21 h p.i. **E.** Biodistribution of ^64^Cu-AAV9 (%ID/g) in left and right (FUS) hemispheres of FUS-treated AAV9-injected (n = 5) and no-FUS AAV9-injected (n = 3) C57BL/6 mice at 22 h p.i. **F**. Reverse transcription quantitative polymerase chain reaction (RT-qPCR) assessment of ssDNA (PKM2) in brain hemispheres of FUS-treated AAV9-injected (n= 4) and no-FUS AAV9-injected (CTL, n= 3). **G.** Correlation between ssDNA genome copies (PKM2) delivered and radiolabeled capsid uptake (%ID/cc). **Abbreviations**: %ID/g: Percent injected dose per gram of tissue. %ID/cc: Percent injected dose per cubic centimeter. C-L: contra-lateral. FUS: focused ultrasound. LH: left hemisphere. RH: right hemisphere. MBs: microbubbles. IV: intravenous injection. PAM: passive acoustic mapping. Brown-Forsythe and Welch ANOVA with multiple comparison correction was performed for the statistical analysis between groups and a paired *t* test compared the mean values between the FUS-treated and contralateral hemisphere in the same group. *p*-values are indicated in the panels.

With the optimal FUS parameters, we then evaluated blood-subtracted ^64^Cu-AAV9 local accumulation within the brain by quantitative PET/CT imaging (*in vivo*) and biodistribution studies (*ex vivo*) in C57BL/6 mice (n = 6) at 21 h and 22 h p.i., respectively (Fig. 3A). The accumulation of radiolabeled viral particles coincided with FUS-target areas and the location of cavitation events in treated subjects. Sliced axial, coronal, and sagittal brain images from PET/CT showed an enhanced accumulation of viral particles in the hippocampal region of the FUS-treated hemisphere (Fig. 3B). From image analysis at the 21 h time point, ^64^Cu-AAV9 spatial-average accumulation within the ROI volume of 10-11 mm^3^ was 0.48 %ID/cc in the mice not receiving FUS. In comparison, in the treated hemisphere of the FUS-treated mice, the mean and maximum accumulation was 2.54 %ID/cc (*p* = 0.015) and 4.54 %ID/cc (*p* = 0.062), respectively (Fig. 3C-D). In the absence of FUS, the AAV9 peak accumulation was ~1 %ID/cc by image analysis (Fig. 3D) and 0.35 %ID/g based on biodistribution (Fig. 3E). The greatest spatial-peak accumulation in the region was 8.42 %ID/cc. Biodistribution following saline perfusion at 22 h after ^64^Cu-AAV9 injection confirmed enhanced accumulation of radiolabeled AAV9 in the entire sonicated hemispheres (Fig. 3E). Despite subject variability, viral accumulation was greater in sonicated hemispheres than contralateral regions, in all cohorts across different studies. Additionally, in a separate study we assessed the correlation between local capsid uptake and ssDNA copies delivered in the sonicated hemisphere (Fig. 3E). Within the same cohort (n_treated_ = 4, n_CTL_ = 3), we quantified ^64^Cu-AAV9 uptake (ROI) 21h post injection and quantify PKM2 ssDNA copies in the FUS-treated hemisphere 22h post injection through PET and qPCR, respectively (Fig. 3G). We observed a 7.5-fold increase in vector genome copies (ΔΔCt) in sonicated hemispheres of treated animals (n_treated_= 4) as compared to hemispheres of non-treated control animals (n_CTL_= 4). Our data obtained points to a correlation (R^2^= 0.9815) between capsid uptake and the number of genome copies delivered (Fig. 3E). Subjects displaying the lowest genome copies in the sonicated hemisphere also exhibited the lowest capsid uptake (%ID/cc).

### FUS enhanced local brain transduction after systemic AAV9 injection

**Figure 4:**
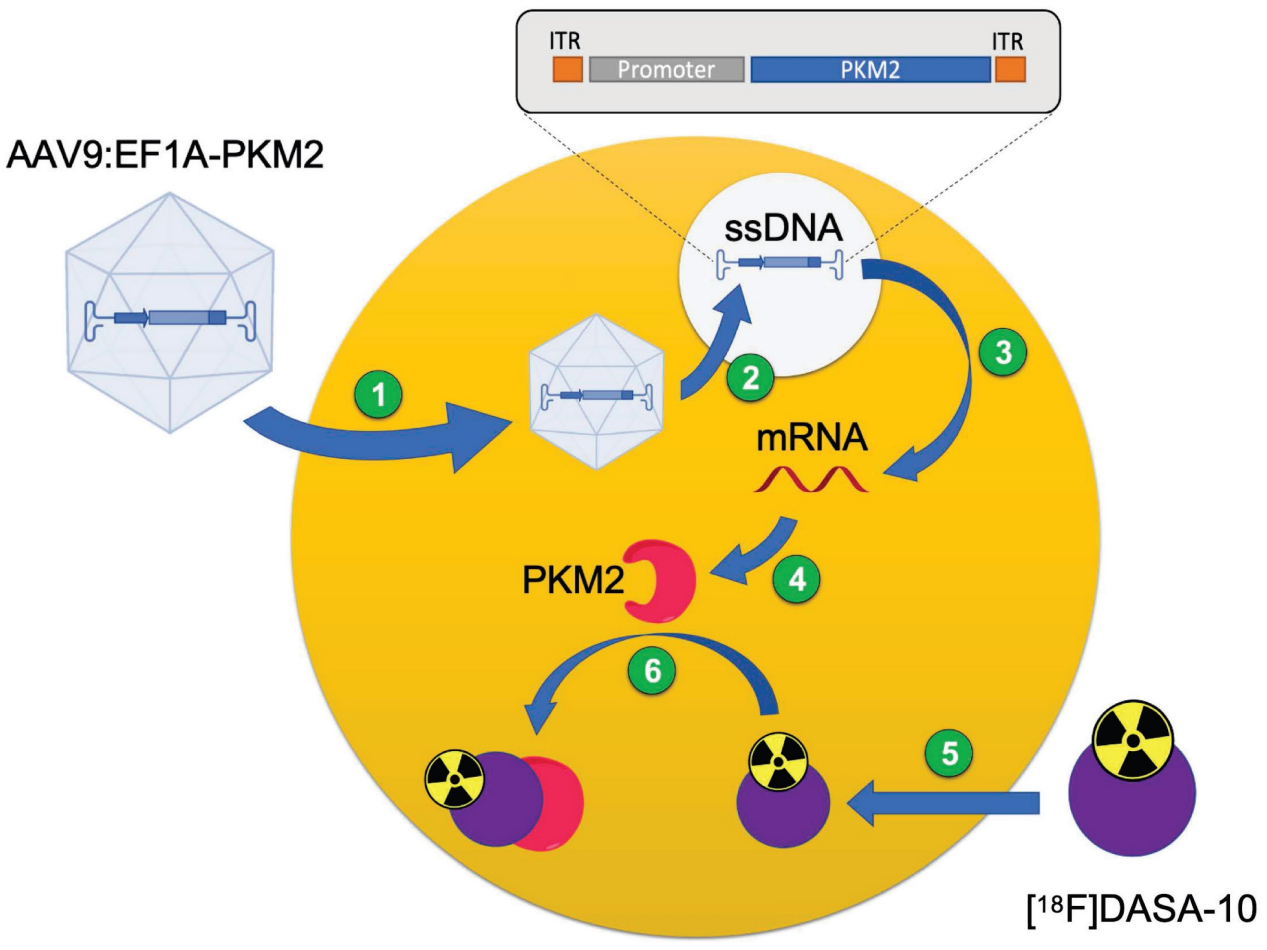
** Schematic illustration of PET reporter gene for gene expression quantification with a PET probe.** 1) AAV9 capsid internalizes into the cell after systemic injection in C57BL/6 mice. 2) Capsid uncages and gene is delivered into the cell. 3) Gene transcription and mRNA synthesis. 4) mRNA translation and protein synthesis (PKM2). 5) PET reporter probe ([^18^F]DASA-10) internalizes into the cell. 6) PET reporter probe reversibly binds to gene-encoded protein for *in vivo* quantification.

**Figure 5:**
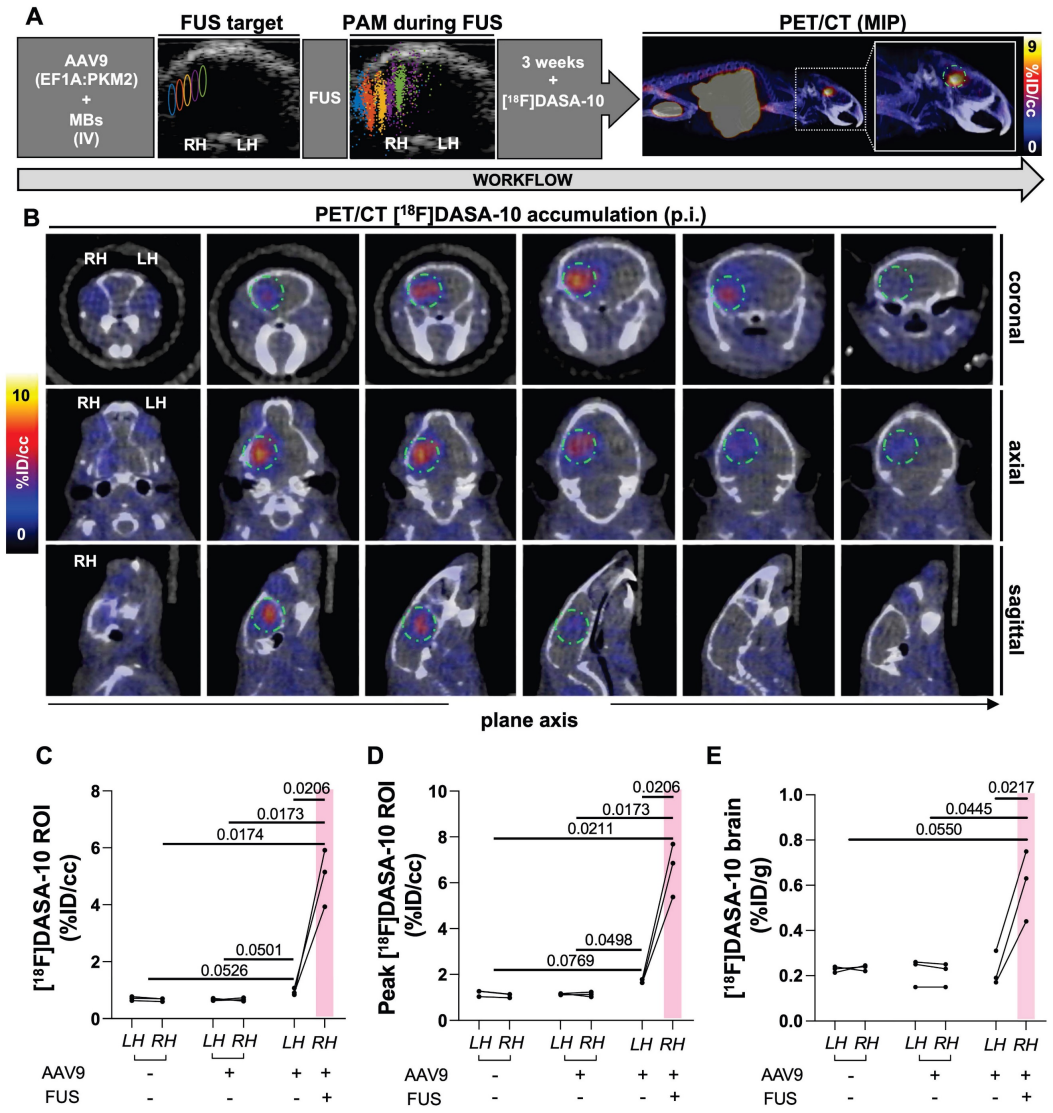
** [^18^F]DASA-10 accumulation study by *in vivo* quantitative PET/CT imaging and *ex vivo* biodistribution analysis 3 weeks after AAV9 injection followed by FUS at 600 kPa. A.** Schematic illustration of workflow, USg-FUS and post-FUS brain cavitation localization area from PAM for mice treated at 600 kPa, maximum intensity projection (MIP) PET/CT image after [^18^F]DASA-10 systemic injection in C57BL/6 mice.** B.** Representative coronal, axial and sagittal plane PET/CT images of an example mouse acquired immediately post systemic injection of [^18^F]DASA-10. Areas of [^18^F]DASA-10 retention have been highlighted with a dotted green circle for clarity. **C.** Image-based blood subtracted radioactivity (%ID/cc) in regions of interest (ROIs) of left and right brain hemispheres of FUS-treated AAV9-injected, no-FUS AAV9-injected and no-FUS no-AAV9-injected groups after injection of [^18^F]DASA-10 (n = 3 per group). **D.** Image-based blood subtracted peak radioactivity (%ID/cc) in brain ROI (10-11 mm^3^) in FUS-treated AAV9-injected, no-FUS AAV9-injected and no-FUS no-AAV9-injected groups after injection of [^18^F]DASA-10 (n = 3 per group). E. Biodistribution post injection (p.i.) of [^18^F]DASA-10 (%ID/g) in left and right hemispheres of FUS-treated AAV9-injected, no-FUS AAV9-injected and no-FUS no-AAV9-injected groups (n = 3 per group). **Abbreviations:** %ID/g: Percent injected dose per gram of tissue. %ID/cc: Percent injected dose per cubic centimeter. LH: left hemisphere. RH: right hemisphere. FUS: focused ultrasound, MBs: microbubbles. IV: intravenous injection. Brown-Forsythe and Welch ANOVA with multiple comparison correction was performed between groups and a paired *t* test compared the mean values between the FUS-treated and contralateral hemisphere in same group. *p*-values are indicated in the panels.

In our previous work on multimodal imaging of AAVs 19, we employed PET reporter gene imaging to monitor the spatial distribution and longitudinal expression of PKM2 in the brain with the [^18^F]DASA-23 PET reporter probe, which is capable of freely crossing the BBB 19, 23, 24. In that study, the transcribed PKM2 mRNA levels were correlated to the PET signal. Here, we systemically administered AAV9 packaging the EF1A-PKM2 transgene and then applied FUS to local areas of the mouse brain. We then assessed the local spatial distribution and transgene expression in the brain, quantified as retention by PET reporter gene imaging (Fig. 4, 5A). Three weeks after the systemic injection of AAV9:EF1A-PKM2 and local FUS treatment, [^18^F]DASA-10 was administered and the AAV9-delivered PKM2 transgene expression was apparent as visualized by dynamic PET imaging. The reporter PKM2 protein expression coincided with the insonified brain areas as compared to untreated areas, where the PET probe signal remained at background levels (Fig. 5B).

From the ROI analysis, the average uptake of [^18^F]DASA-10 was significantly greater in the sonicated brain areas (5.0 %ID/cc), compared to contralateral regions (0.9 %ID/cc, *p* = 0.021) in the same subjects (n = 3) and compared to 0.67 %ID/cc in mice not receiving FUS (*p* = 0.017) (Fig. 5C). Across subjects, the average peak uptake was 6.64 %ID/cc (*p* = 0.021 compared with the contralateral brain areas in the same subjects and *p* < 0.021 when compared with the control groups not receiving FUS) (Fig. 5D). Thus, from the values above, the fold change of the spatial mean and maximum compared to the cohort without FUS was 7.5- and 10.0-fold, respectively (Fig. 5C-D). Reporter gene expression was also greater (*p* = 0.051) in the contralateral brain of mice treated with MBs, AAV9 and FUS than in the same regions of mice treated only with MBs and AAV9 (Fig. 5C). In a control experiment within subjects injected with AAVs without FUS treatment (n = 3) or without AAV dose or sonication (n = 3), significant differences between analogous brain areas were not observed (Fig. 5C-D).

Biodistribution results, assayed after perfusion with saline at 1 h after radiotracer administration, were consistent with the ROI analysis results obtained by PET/CT imaging; a larger [^18^F]DASA-10 accumulation in sonicated areas of the brain as a result of greater PKM2 expression (Fig. 5E) was observed. [^18^F]DASA-10 retention in the entire insonified hemisphere was ~0.6 %ID/g in mice receiving AAV9 packaging the EF1A-PKM2 transgene and subjected to lateral FUS treatment (n = 3) compared with ~0.2 %ID/g in mice receiving AAVs without FUS. Within the control groups, mice receiving only AAVs (n = 3) and those receiving neither AAV nor FUS treatment (n = 3) did not exhibit a significant accumulation increase in analogous brain regions (Fig. 5E).

### Radiolabeled capsid accumulation and transduction colocalized in analogous brain areas within the same cohort

**Figure 6:**
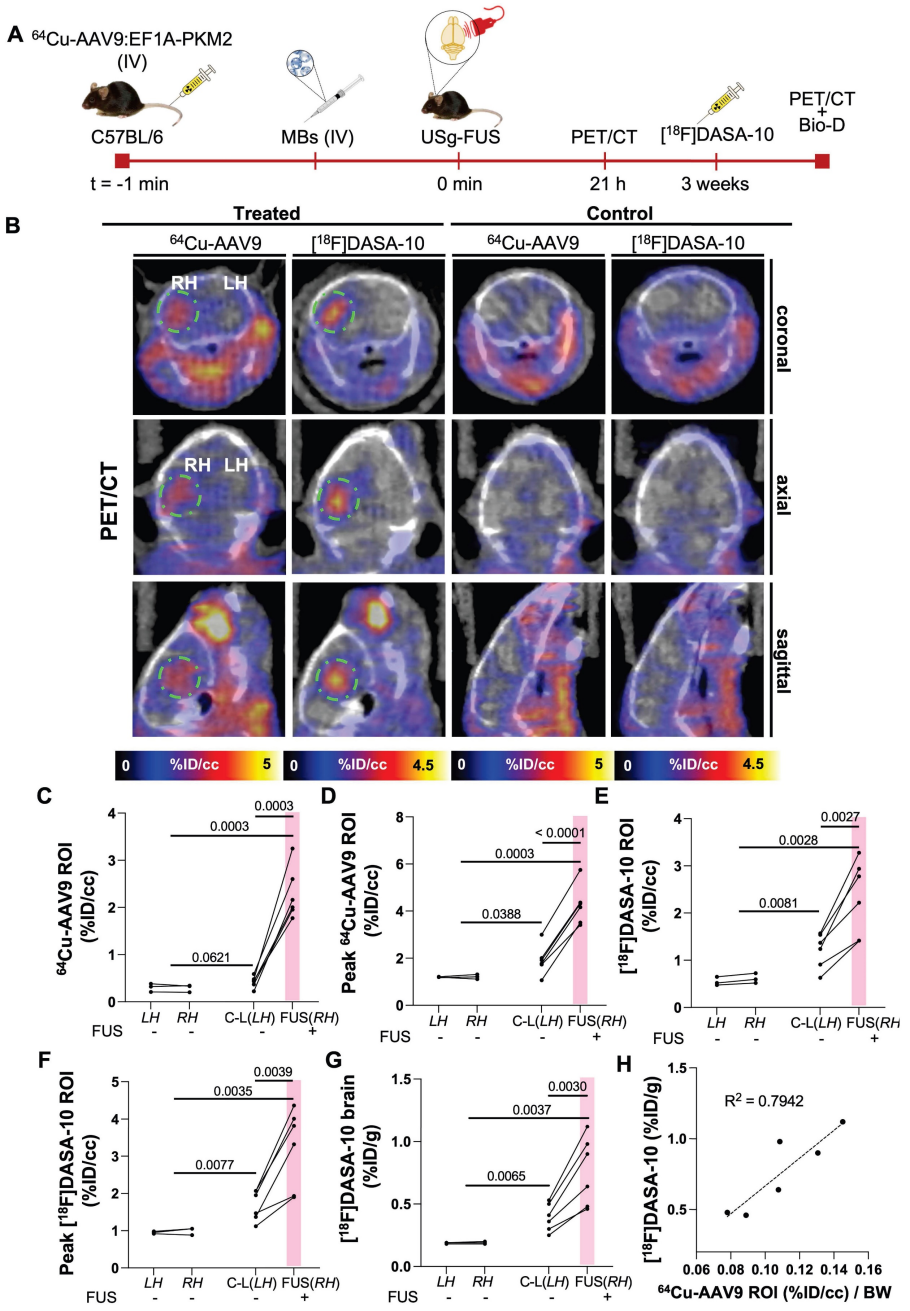
** Assessment of ^64^Cu-AAV9 accumulation and PKM2 transduction within the same cohort. A.** Schematic illustration of FUS-assisted ^64^Cu-AAV9:EF1A-PKM2 delivery for assessment of uptake and transgene expression, 21 h and 3 weeks after systemic capsid administration, respectively, within the same C57BL/6 mouse cohort. **B.** Representative PET/CT images for ^64^Cu-AAV9 accumulation and PKM2 expression with [^18^F]DASA-10. Areas of ^64^Cu-AAV9 accumulation and [^18^F]DASA-10 retention have been highlighted with a dotted green circle for clarity. **C.** Image-based blood subtracted radioactivity (%ID/cc) in the brain ROI (10-11 mm^3^) of FUS-treated AAV9-injected (n = 6) and no-FUS AAV9-injected (n = 3) C57BL/6 mice at 21 h post injection (p.i.) of ^64^Cu-AAV9.** D.** Image-based blood subtracted peak radioactivity (%ID/cc) in brain ROI (10-11 mm^3^) of FUS-treated AAV9-injected (n = 6) and no-FUS AAV9-injected (n = 3) C57BL/6 mice at 21 h p.i. of ^64^Cu-AAV9. **E.** Image-based blood subtracted radioactivity (%ID/cc) in brain ROI (10-11 mm^3^) in FUS-treated AAV9-injected (n = 6) and no-FUS AAV9-injected (n = 3) C57BL/6 mice immediately after injection of [^18^F]DASA-10. **F.** Image-based blood subtracted peak radioactivity (%ID/cc) in brain ROI (10-11 mm^3^) of FUS-treated AAV9-injected (n = 6) and no-FUS AAV9-injected (n = 3) C57BL/6 mice after injection of [^18^F]DASA-10. **G.** Biodistribution (Bio-D) p.i. of [^18^F]DASA-10 (%ID/g) in the left and right hemispheres of FUS-treated AAV9-injected (n = 6) and no-FUS AAV9-injected (n = 3) C57BL/6 mice. **H.** Correlation between ^64^Cu-AAV9 accumulation and [^18^F]DASA-10 retention in brains of FUS-treated AAV9-injected (n = 6) subjects. **Abbreviations**: %ID/g: Percent injected dose per gram of tissue. %ID/cc: Percent injected dose per cubic centimeter. C-L: contra-lateral. LH: left hemisphere. RH: right hemisphere. FUS: focused ultrasound, MBs: microbubbles. IV: intravenous injection. BW: body weight. Brown-Forsythe and Welch ANOVA with multiple comparison correction was performed between groups and a paired *t* test compared the mean values between the FUS-treated and contralateral hemisphere in the same group. *p*-values are indicated in the panels.

In order to evaluate co-localization of the capsid and transduction, we then evaluated both radiolabeled-capsid accumulation and longitudinal transgene expression within the same cohort of mice (n = 6) (Fig. 6A-B). We observed ^64^Cu-AAV9:EF1A-PKM2 accumulation and, ultimately, [^18^F]DASA-10 retention in the FUS-target areas of the mouse brain. Without FUS treatment, capsid accumulation averaged 0.29 %ID/cc in mice treated with AAV only and 0.42 %ID/cc (*p* = 0.0621) in the contralateral brain of FUS-treated mice (Fig. 6C). With FUS treatment, mean and maximum capsid accumulation reached 2.29 and 4.25 %ID/cc within the ≈10 mm^3^ ROI in insonified brain regions (Fig. 6C-D), an increase of 14-fold over the no-FUS control mice. Three weeks after ^64^Cu-AAV9 administration, [^18^F]DASA-10 uptake in insonified brain areas reached an average spatial maximum of 3.22 %ID/cc, compared to 0.58 %ID/cc in the non-insonified controls (Fig. 6E-F). Further, ^64^Cu-AAV9 uptake (0.42 %ID/cc, *p* = 0.062) and [^18^F]DASA-10 retention (1.21 %ID/cc, *p* = 0.008) in the contralateral side of the FUS-treated brain were also greater than the observed uptake and retention (^64^Cu-AAV9: 0.29 %ID/cc, [^18^F]DASA-10: 0.55 %ID/cc) in the brain of the no-FUS-treated mice (Fig. 6C, E). These trends are consistent with radiotracer accumulation evaluated by biodistribution studies in the entire brain assessed 1 h after PET/CT image acquisition and blood perfusion (Fig. 6G). Our data point to a spatial correlation between capsid accumulation delivered upon temporal FUS-assisted BBB disruption and local transgene expression in treated areas. Accumulation and transgene expression outside of the insonified region were also assessed through PET imaging and quantitation (%ID/cc), and off-target differences were not detected between treated (FUS, RH) and non-treated subjects (Supplementary Fig. S7).

In addition to imaging PKM2 expression via PET, we also validated the mRNA concentration. We performed a reverse transcription quantitative polymerase chain reaction (RT-qPCR) and Western blotting of mouse brain tissue (Supplementary Fig. S8) to evaluate PKM2 mRNA and protein expression levels, respectively. Systemic injection of AAV9 packaging the EF1A-PKM2 transgene followed by FUS in one hemisphere resulted in an average 102-fold increase in mRNA concentration (*p* = 0.0036) in the treated hemisphere compared with the no-FUS control cohort. mRNA in the insonified hemisphere was 12.5-fold higher than in the contralateral hemisphere (*p* = 0.0020) (Supplementary Fig. S8B). PKM2 mRNA was translated to PKM2 protein 3 weeks post-capsid injection, as detected by Western blot, in the FUS-treated hemispheres (Supplementary Fig. S8A). Enhanced PKM2 protein expression observed in sonicated areas of the brain correlated with our findings for quantitative PET reporter gene imaging. As demonstrated in our previous work, exogenous PKM2 expression after AAV9-mediated transgene delivery did not increase the mRNA level of the translocator protein (TSPO) inflammatory marker in brains transduced with AAV9:EF1A-PKM2 (Supplementary Fig. S8C) 19.

We also assessed the correlation between PET readouts for ^64^Cu-AAV9 accumulation (%ID/cc) through PET and transgene expression, quantified as [^18^F]DASA-10 retention, by gamma counting biodistribution analysis (Figure 6H). ^64^Cu-AAV9 (%ID/cc) and [^18^F]DASA-10 uptake are correlated across animals in this study (R^2^= 0.7942). Within the relatively small mouse brain, small differences in the localization of the ultrasound beam and small differences in the skull thickness and properties are anticipated to limit the correlation among animals. Capsid accumulation and transgene expression, which depend on the AAV serotype tropism, also differ spatially within the brain due to tissue specificity and cell density in the target areas.

### Fluorescence imaging of the spatial distribution of transduction after FUS-assisted AAV delivery

**Figure 7:**
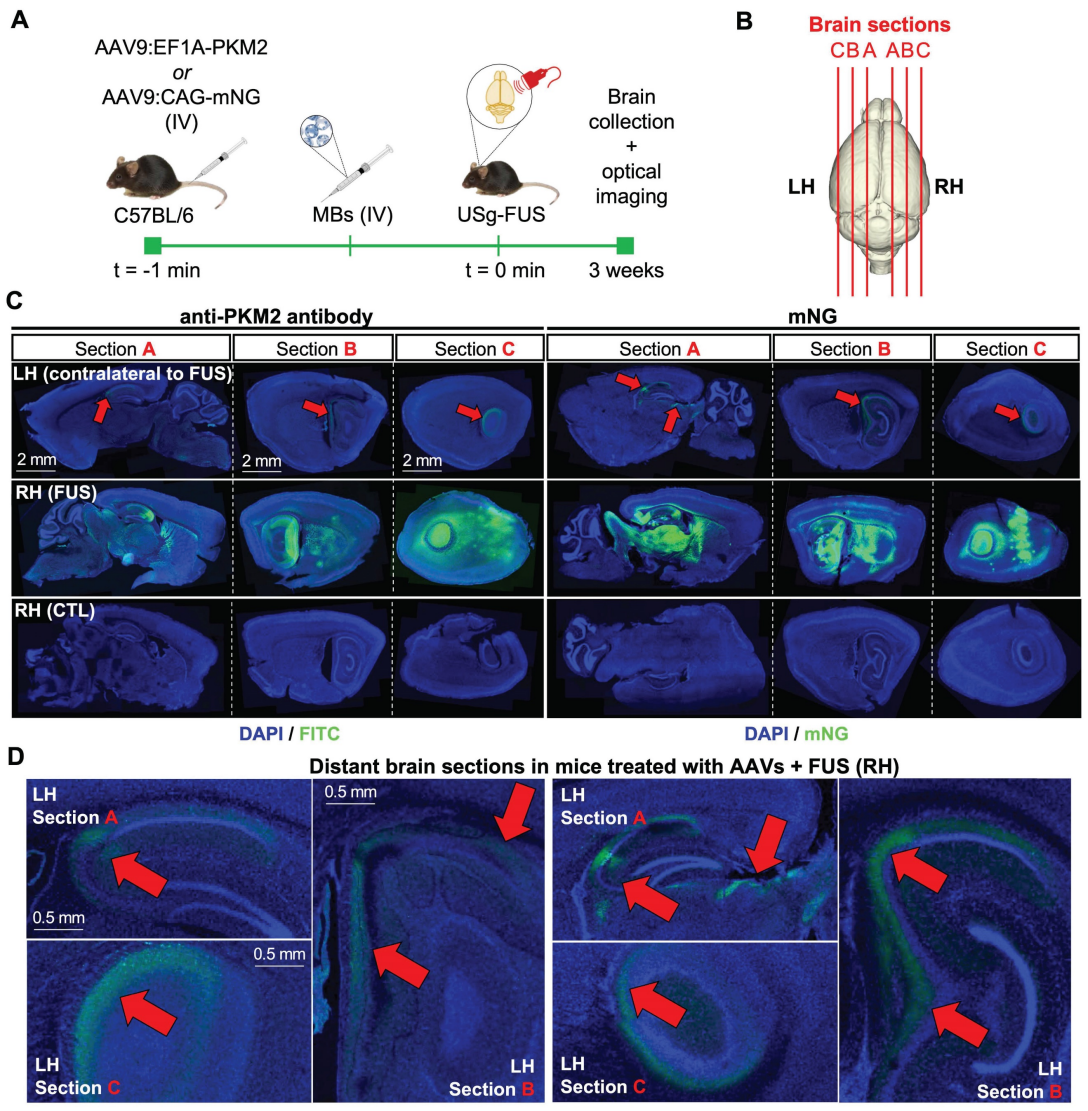
** Assessment of transduction of PKM2 and mNeonGreen (mNG) reporter genes by optical imaging three weeks after capsid injection. A.** Schematic illustration of FUS-assisted delivery of AAV capsids encapsulating PKM2 or mNG reporter genes for transduction assessment with optical imaging in C57BL6 mice. **B.** Schematic illustration of brain sectioning for optical imaging. **C.** Fluorescence microscopy images of brain slices (100 μm) of the FUS-treated and no-FUS AAV9-injected control (CTL) mice. Assessment of PKM2 transduction through anti-PKM2 protein staining (DAPI/FITC) or mNG fluorescence detection (DAPI/mNG). **D.** Magnified regions of slices of the left hemisphere of brains receiving FUS treatment in the right hemisphere. **C-D.** Red arrows indicate locally-enhanced gene expression. **Abbreviations:** LH: left hemisphere. RH: right hemisphere. FUS: focused ultrasound, MBs: microbubbles. IV: intravenous injection, CTL: control.

To further explore the spatial distribution of the AAV delivery of transgenes and protein expression in the brain at high magnification, we included fluorescent transgenes in our capsid and administered the AAV together with FUS treatment. The spatial distribution of the PKM2 and mNeonGreen (mNG) transgenes at 3 weeks was then assessed after systemic AAV9 administration following MB-assisted FUS treatment in C57BL/6 mouse brains (Fig. 7A-B). Gene expression for the EF1A (used with PKM2) and CAG (used with mNG) promoters within the same capsid was consistent and should therefore be comparable, as discussed in 19. Employing a 10× magnification, we analyzed microscope images of brain slices of mice injected with AAV9:EF1A-PKM2 and observed PKM2 expression in sonicated areas (Fig. 7C, left panels) displaying the fluorescence intensity in the hippocampal and surrounding regions. The findings were similar for AAV9:CAG-mNG treatment (Fig. 7C, right panels). We found that sonicated local areas of the brain displayed the highest mNG fluorescent-protein signal. Notably, low levels of PKM2 and mNG expression were observed in the contralateral regions to the FUS treatment, near the hippocampus, and only in mice treated with both FUS and MBs (Fig. 7D). Overall, our results support previous findings for PET reporter gene imaging studies showing increased zonal transgene transduction in sonicated areas of the brain after capsid delivery. The results also suggest a low level of transport of AAVs from the treated region to the distant brain.

## Discussion

The increasing number of preclinical studies and clinical trials employing AAVs as the leading gene therapy delivery platform reflects the extraordinary potential of these viral vectors for efficient gene transduction, particularly relevant to treatment of rare diseases. Remarkable progress in the field has been made in recent years, including the development of novel engineered AAV variants (i.e., PHP.eB, CAP-B10) displaying wide cell-type specific brain transduction, as opposed to naturally-occurring serotypes that exhibit limited BBB transport (i.e., AAV9, AAV2, AAV5) 7, 8, 30. Genetic intervention of CNS disorders is currently limited to the treatment of spinal muscular atrophy (SMA) in children employing an FDA-approved AAV9-based therapy, marketed as Zolgensma^®^ 31.

We previously used multimodal imaging to assess the accumulation and longitudinal transgene expression of novel engineered AAV variants *in vivo* 19. However, these capsids are not suitable for targeted gene delivery, as they can freely cross the BBB and widely transduce brain cells. In this work, we use AAV9 22, a naturally-occurring serotype which has limited transport across the BBB, displays an inherent hepatotropism and has shown promise in treating several conditions, including SMA 31, Huntington's disease 32, and Rett syndrome 33. Intracranial injection has been employed for AAV delivery into the brain, as it allows for precise targeting of specific brain regions; however, this technique is invasive and there is a potential risk of tissue damage and adverse effects 34. We have previously explored the biodistribution and pharmacokinetics of AAVs in wild type mice without ultrasound in three papers 19-21. The offtarget biodistribution is not significantly changed by the application of ultrasound to a small region of the brain as demonstrated by PET imaging and ROI quantification (Supplementary Fig. S7). For reference, the typical accumulation assessed by biodistribution in blood, heart, lungs, liver, spleen, kidneys, stomach, intestine, muscle and bone are 16.9±3.6, 3.4±0.5, 7.1±0.9, 18.3±1.2, 15.8±3.3, 5.6±0.8, 4.2±0.2, 7.8±0.9, 0.8 ±0.1 and 2.8±0.6 %ID/g, respectively 21. We note that AAV9 is still circulating at the 21 hour time point and additional signal within the brain results from the presence of circulating AAVs.

In recent years, the incorporation of ultrasound has shown efficacy in enhancing AAV delivery into the murine brain. Preclinical studies have demonstrated that non-invasive systemic (IV) 10, 15 or intranasal administration (IN) 14, 35 of AAVs in combination with MB-FUS treatment can enable efficient and stable transgene expression in target areas of the brain that is comparable to invasive intracranial injections without significant side-effects. However, to date, quantification of transduction has been exclusively assessed employing invasive techniques 12, 36. Such quantitation is important since achieving therapeutic levels of transduction in the CNS depends on the receptor binding affinity for each serotype and the receptor density in the target area 36. In this context, PET imaging is a valuable method to non-invasively monitor biodistribution and tissue-specific accumulation of radiolabeled AAV9 vectors in real-time. This is significant since imaging can provide valuable insights into the distribution patterns throughout the body and unintended interactions with tissues or organs beyond the targeted site.

We employed an ultrasound-guided MB-FUS treatment to generate a transient BBB disruption of local areas of the mouse brain through cavitation of lipid-shelled MBs intravenously administered to increase BBB permeability in target areas, and monitored AAV9 transport and transduction with PET. While low levels of capsid uptake were observed in both hemispheres in the control groups, PET allowed us to confirm that ^64^Cu-AAV9 accumulation was significantly enhanced by FUS treatment at 600 kPa. Accumulation increased with increasing acoustic pressures in the sonicated hemispheres compared to the non-sonicated hemispheres of treated mice. While the highest ^64^Cu-AAV9 uptake occurred at 740 kPa, undesirable red blood cell extravasation occurred, and therefore 600 kPa was used in this study. These results agreed with power spectrum comparisons between pressure groups which showed enhanced broadband levels at 740 kPa.

Here, we used 1.5 MHz ultrasound to minimize the brain region transduced in mice; however, in order to facilitate the translation of this methodology into the clinic, frequencies in the range of 0.5 MHz will be tested. The ~11 mm^3^ volume that presented the highest PET radioactive intensity signal was similar to the theoretical FUS-treated volume (~15.6 mm^3^) as defined by the technical FUS parameters employed. Compared to the PHP.eB and CAP-B10 AAV variants, which exhibited capsid accumulation throughout the brain (12-14 %ID/cc) in 19, FUS allowed us to deliver spatial peak AAV9 doses up to 8.42 %ID/cc with an average maximum accumulation within an ~10 mm^3^ ROI across mice of ~4 %ID/cc. This trend was also confirmed by the data from *ex vivo* biodistribution studies.

On PET reporter gene imaging, we observed an increase in protein expression in sonicated hemispheres of the brain 3 weeks after treatment with AAV9 and ultrasound. In previous work without the application of ultrasound, AAV9 delivery was not sufficient to result in an enhancement in the PET reporter gene. In this work, we achieved [^18^F]DASA-10 retention between 3 and 6 %ID/cc 3 weeks p.i. of AAV9. Thus, the data points to AAV9 transduction efficiency in sonicated regions that is similar to PHP.eB (∼4 %ID/cc), a variant that exhibits wide brain transduction across the entire brain 7.

The spatial distribution of ^64^Cu-AAV9 accumulation in the mouse brain was similar to that of the AAV9-delivered reporter protein expression, with the highest PET signal observed typically near the targeted hippocampal and thalamus regions of sonicated hemispheres. In our previous work, we have observed zonal variations in reporter protein intensity compared to capsid accumulation. These differences might be associated with cell density and cellular specificity, where the receptor-binding affinity of the AAV serotype may impact spatial distribution and transduction efficiency 36. In the brain, more than 80% of cells transduced after AAV9 administration were neurons and astrocytes 37.

Interestingly, we observed an enhancement in ^64^Cu-AAV9 accumulation (*in vivo*) and [^18^F]DASA-10 retention (*in vivo* and *ex vivo*) in the non-sonicated hemispheres (*C-L*(LH)) of FUS-treated mice compared to the matching brain hemispheres (LH) of untreated mice, within the same cohort, through PET imaging. These results correlated with data from optical imaging, which showed transgene expression in non-sonicated hemispheres of the brain (LH), specifically around the outer shell of the hippocampus, which was obvious even in the most distant brain sections from the FUS focus. Other studies have also suggested that AAVs can be transported within the brain, typically moving along perivascular bundles 38 and that uptake of large and small molecules can be enhanced within the brain by ultrasound without microbubbles 39. A further advantage of the optical and PET reporters used here is the small changes in the contralateral brain are mapped and quantified.

Traditionally, mRNA/DNA titers have been employed to assess AAV-transgene expression 16, 36. However, these invasive methodologies may not always provide an accurate reflection of transgene expression, as they do not consider post-transcriptional regulatory mechanisms such as mRNA/DNA stability, translation efficiency, or protein degradation. Our data supports the ability of PET imaging to non-invasively assess expression of the transgene.

The hippocampus is the target region for many proposed AAV trials and therefore is a particularly important target. In mice, the hippocampus is located in the medial temporal lobe of the brain and is responsible for various cognitive functions such as spatial navigation, memory consolidation and learning, and has a similar structure and function as that in humans. With image-guided FUS-BBB methods, this region can be selectively insonified. Our 2D therapeutic array facilitates guidance of the beam in three dimensions. We monitored the location and efficacy of the insonation in real-time by passive cavitation detection and passive acoustic mapping (PAM) 28. This is important, as stable cavitation can enhance delivery without tissue damage. On the contrary, inertial cavitation can induce damage and occurs when the pressure exceeds a threshold and the MBs rapidly expand and collapse, generating shockwaves. In our study, analysis of PAM data after FUS treatment showed that areas receiving the highest density of cavitation events colocalized with the US-guided targeted area.

The MB dose employed in this work (∼5×10^6^ MB for a 20 g mouse = ∼2.5×10^8^ MB/kg) was within the range reported in 36 (~1 x 10^8^ MB/kg) and that reported in 14, 35 (∼8×10^8^ MB/kg), and is comparable to the recommended dosage for the clinical-grade MB Definity® (∼1.2×10^8^ MB/kg). We confirmed no damage to blood vessels upon FUS treatment by H&E analysis and T2*-weighted MRI 29. We also evaluated quantified translocator protein (TSPO) mRNA, a marker that is associated with neuroinflammation, and confirmed that exogenous PKM2 expression in the brain resulting from AAV9 transduction did not increase TSPO mRNA levels.

Disparities were observed within mice treated at the same acoustic pressure. We hypothesize that variations in capsid accumulation within cohorts may be due to a finite spatial selectivity during the FUS treatment, which is subjected to technical limitations in the size of the transducer array and the reduced volume of the murine brain. These handicaps may be overcome in translation to humans due to a larger size of the brain, achieving an accuracy capable of targeting specific subnuclei and subregions 40. An additional factor that might impact transduction between studies is the capsid full/empty ratio resulting from the AAV manufacturing process in cell culture affording “empty” capsids, which lack the vector genome and cannot provide therapeutic benefits 41. Based on quality control data, the vectors used in this work had a full/empty capsid ratio greater than 80%. Some studies suggest that empty AAV capsids might improve gene transfer by addressing the issue of pre-existing humoral immunity to AAV 42 and this question will be addressed in future work.

## Conclusion

In summary, combining microbubble and focused ultrasound treatment generated a locally transient disruption of the blood-brain barrier, significantly increasing its permeability and resulting in enhanced transport of systemically administered AAV9 into the murine brain. PET imaging allowed us to non-invasively quantify viral vector accumulation in FUS target areas. PET reporter gene imaging quantified transgene expression *in vivo*, and optical imaging further confirmed zonal transduction of viral particles in sonicated areas across the brain. Technologies capable of non-invasively assessing and monitoring the delivery of therapeutic gene doses to specific areas of the brain are critical for the translation of AAV vector-based therapies into the clinic.

## Methods

### Materials & reagents

The detailed list of materials and reagents is provided in the Supplementary information.

### AAV production

AAV9 was produced as described in our previous work 19. Briefly, AAVs were harvested 5 days after triple transfection in HEK293 cells by PEG precipitation of 3- and 5-days media and osmotic lysis of cell pellets. Crude AAVs were then purified by extraction from iodixanol density gradients and buffer exchanged into Dulbecco's phosphate-buffered saline (DPBS). Viral titers were determined by qPCR on a woodchuck hepatitis virus post-transcriptional regulatory element (WPRE) present in all packaged AAV genomes as detailed below.

### Radiolabeling of AAVs

All radiolabeling experiments were conducted under the Controlled Radiation Authorization (CRA) approved by Stanford University (Palo Alto, CA). Radiolabeling of AAVs was performed using our previously reported method 21. [^64^Cu]CuCl_2_ was produced at the MIR Cyclotron Facility at Washington University School of Medicine. The stock solution of AAV9 (4x10^12^ vg) in 1xPBS (0.2 mL) was mixed with aqueous Na_2_CO_3_ (0.1 M, 20 μL, pH = 9.2), 2 mM tetrazine-PEG_5_-NHS (Tz-PEG_5_-NHS, 2 nmol, 1 μL in DMSO) and incubated at 25 ºC for 30 minutes. After addition of 1xPBS (0.1 mL), the mixture was transferred to a mini-dialysis device (20 kDa molecular weight cut-off (MWCO)), dialyzed in 1xPBS (15 mL) for 4 hours and subsequently transferred for overnight dialysis (500 mL). Dialyzed Tz-AAVs were collected and further incubated over a period of 0.5-1 hours with ^64^Cu-(NOTA)_8_-TCO. The latter was freshly prepared from the reaction of [^64^Cu]CuCl_2_ (111-148 MBq (3-4 mCi), 4-5 μL) and 10 μM (NOTA)_8_-TCO (40-100 pmol, 4-10 μL) in ammonium citrate buffer (20 μL, pH 6.5) at room temperature. The incorporation of Cu-64 to (NOTA)_8_-TCO was complete after 30 minutes as monitored by instant thin-layer chromatography (iTLC). After radiolabeling, EDTA (0.5 M, 3 μL) was added to the mixture and allowed to react for a further 15 minutes. Radiolabeled AAVs were purified employing a 100 kDa MWCO centrifugal filter unit (Thermo Fisher Scientific) with 1xPBS (3x15 mL) containing 0.001% Pluronic F-68 (Gibco) and concentrated in ~200 μL volume.

### Animal models

All animal experiments were conducted with a protocol approved by the Administrative Panel for Laboratory Animal Care (APLAC) at Stanford University. AAVs were evaluated in wild-type 8-12 week-old C57BL/6 (Charles River) mice.

### USg-FUS treatment and passive acoustic mapping (PAM)

USg-FUS treatment was performed with a programmable ultrasound system (Vantage 256, Verasonics, Kirkland, VA, USA) enabling control of the FUS treatment and real-time ultrasound guidance and monitoring. We employed a 1.5-MHz 128-element array allowing electronic steering (Imasonic, Voray sur l'Ognon, France) with a focal depth of 55 mm and a -6 dB focal dimension of 2.7 mm (axial) × 0.7 mm (transverse) × 0.4 mm (transverse) (Supplementary Fig. S1). The array was specifically developed for FUS treatments in rodents 43. The animal was placed in the supine position with its head held by a stereotaxic frame designed in-house and attached to a 3D stage for fine positioning. The target (hippocampal) ROI in the right hemisphere was identified by US guidance based on the outline of the skull shape which was compared to anatomical annotations from the Allen Mouse Brain Atlas and Allen Reference Atlas 44. In test experiments, we looked at Gd contrast uptake with MRI to validate and confirm the intended target was sonicated resulting in successful BBB opening. After identifying the ROI within the hippocampal region of the right hemisphere under ultrasound guidance, FUS sonication started 10 s after the tail vein microbubble injection. A second array (L12-5) inserted into the center of the therapeutic array) applied B-mode pulses (for localization) at 10.4 MHz, 1 cycle, 136 kPa peak negative pressure (PNP) (MI = 0.04) and contrast pulse sequencing (CPS) (right before injection and during FUS treatment) at 5.2 MHz, 1 cycle, 250 kPa PNP (MI = 0.11). Each pulse was measured with a calibrated needle hydrophone (HNP-0400, Onda, Sunnyvale, CA) in degassed water.

During the 1.5 MHz treatment, anesthesia was maintained using isoflurane and pure O_2_. In the validation experiments, the PNP was set to 420, 600 or 740 kPa, respectively, as measured in water with a calibrated needle hydrophone (HNP0400, Onda, Sunnyvale, CA, USA). As the 600 kPa pressure yielded the highest AAV accumulation with no apparent red blood cell extravasation as confirmed through H&E staining, we selected this pressure to study AAV accumulation following BBB opening. For the mouse skull, the insertion loss reported by other groups at 1.5 MHz is ~20 % (18.1% for 45, 20% for 46). We measured the insertion loss at 1.5 MHz from one mouse skull specimen with a needle hydrophone (HNP-0400, Onda Corp.) and similarly found a 20% reduction in pressure. Therefore, the estimated derated PNP in the brain is estimated to be 336, 480 and 592 kPa for a measured PNP in free field of 420, 480 and 740 kPa, respectively.

The FUS array has a small f-number yielding a small focal volume for precise targeting inside the murine brain which results in a decrease in acoustic pressure when steering the beam away from the geometric focus when the driving voltage is kept constant. Although the pressure change is small for the 5x5 grid sequence tested in this work, we implemented a compensation in the driving signals to maintain the same pressure for all grid points. Compensation factors were automatically estimated based on 3D simulations of the pressure field realized with FOCUS 47 (Supplementary Fig. S9). Note that the implementation of the pressure compensation only relates to the change in pressure when the beam is steered and does not consider the effect of the skull or tissue.

Non-targeted lipid-shelled MBs produced in-house (distearoylphosphatidylcholine (DSPC):1,2-distearoyl-sn-glycero-3-phosphoethanolamine-N-[maleimide(polyethylene glycol)-2000 (DSPE-PEG2000); 90:10 molecular ratio) were activated after shaking prepared vials for 45 s (Vialmix). We performed size isolation with centrifugation to increase the proportion of MBs with a diameter >2.5 μm, yielding a median diameter of 4.5 µm. For a 20 g mouse, the MB dose was a 50 µL bolus of 5×10^6^ MBs (2.5×10^8^ MB/kg). With the imaging transducer, radio frequency (RF) signals were passively recorded during the FUS sonication for the 5 spatial positions aligned with the imaging plane. Real-time processing was implemented to display both the spectrum of the receive echoes and the passive acoustic maps (PAM) following the angular spectrum approach 28. We processed the PAM in three different bandwidths: harmonics, ultra-harmonics and broadband. The 4th to 8th harmonics (i.e., 4.5, 6, 7.5, 9 and 10.5 MHz) were utilized to reconstruct the PAM with a bandwidth of 0.2 MHz and assumed to correspond to stable cavitation. The position of the maximum for each PAM map for each sonication was compared to the set position of the focal beam. All processing was implemented in Matlab (r2020b, Mathworks, Natick, MA, USA) to work in real-time within the Verasonics Matlab-based software interface. To quantify the level of stable and inertial cavitation we compared the power spectrum in the harmonics band (centered at 4.5, 6, 7.5, 9 and 10.5 MHz; 0.2 MHz bandwidth) and the broadband (centered at 5.25, 6.75, 8.25 and 9.75; 1.0 MHz bandwidth) (Figure 2G). The power spectrum *PS_F_* for a frequency band *F* was calculated as:



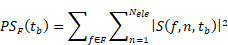



where *S(f,n,t_b_)* is the Fourier transform of the RF signal for the element *n* of the imaging array at the treatment time *t_b_*. *F* represents either the harmonics or the broadband frequencies. We then averaged the power spectra among animals in the same pressure group and among the five recorded focus positions for each animal:



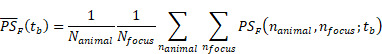



### Magnetic resonance imaging (MRI)

MRI was performed using a Bruker 11.7 Tesla small animal scanner (Bruker BioSpin MRI, Ettlingen, Germany) equipped with a cross coil configuration with a mouse body resonator for transmit and a mouse surface coil for receive. Images were acquired using ParaVision 360 (Bruker BioSpin MRI). Permeability of the BBB was determined with a T1 weighted (T1w) sequence (2D RARE sequence, RARE factor = 2, repetition time (TR) 250 ms, echo time (TE) 6.7 ms, 1 mm slice thickness, 1 mm interslice distance, 13 images, field of view (FOV) = 2 x 2 cm, matrix = 384 x 384, number of acquisitions (NA) = 6) following intravenous injection of Gd-HPDO3A (Prohance®, 0.5 μmol/g mouse body weight). Hemorrhage was (2D FLASH sequence, flip angle (FA) = 15º, repetition time (TR) 250 ms, echo time (TE) 15 ms, 1 mm slice thickness, 1 mm interslice distance, 13 images, field of view (FOV) = 2 x 2 cm^2^, matrix = 192 x 192, number of acquisitions (NA) = 4).

### PET/CT imaging and biodistribution (BioD)

For the FUS optimization (Fig. 2) and capsid accumulation studies (Fig. 3, 6), ^64^Cu-AAV9:EF1A-PKM2 (2×10^11^ vg, ∼5-12 μCi/mouse) was systemically injected into C57BL/6 mice placed on a custom 3D-printed mouse bed in the Siemens Inveon PET/CT scanner immediately after the start of the workflow protocol for image acquisition. Images were obtained at 21 h post capsid injection.

For monitoring of PKM2 gene expression with [^18^F]DASA-10 (Fig. 5-6), AAV9:EF1A-PKM2 (5×10^11^ vg) and ^64^Cu-AAV9:EF1A-PKM2 (2×10^11^ vg) were systemically injected into C57BL/6 mice, respectively. At 3 weeks post injection (p.i.) of the AAV capsid, [^18^F]DASA-10 (SA= 4315.4 ± 2737.4 mCi/µmol) was administered via tail injection (∼100 μCi/mouse). PET/CT dynamic scanning was performed for 30 minutes, and the 30-minute accumulation was reported. [^18^F]DASA-10 production was carried out at the Stanford CRF 25. The list mode data acquired for PET scan (30 minutes, dynamic) was reconstructed and analyzed as described in the *PET/CT image analysis* section.

### PET/CT image analysis

PET data were acquired in 30-min list mode at 0 h for [^18^F]DASA-10 and 21 hours for ^64^Cu-AAV9. Raw list mode data were reconstructed using 3D ordered-subset expectation maximization using maximum aposteriori (3D-OSEM/MAP) image reconstruction and converted to units of percent injected dose per cubic centimeter (%ID/cc). For dynamic image analysis of [^18^F]DASA-10, the 30-min list mode data was segmented into 20 static time frames (15 x 8, 60 x 8, 300 x 4; seconds x frames) and reconstructed as stated above. Quantitative PET image analysis was performed with Inveon Research Workplace (IRW) software after the co-registration of PET and CT images. Images were quantified by manually drawing ROI in the FUS treated area. AAV uptake value subtracted blood radioactivity was calculated as previously reported in 19 using 4% brain vascular volume fraction and the blood activity was estimated based on a ROI placed in the cardiac chamber.

### Autoradiography for biodistribution analysis

Brain tissue was collected 22 h post radiolabeled-AAV9 injection, sectioned into 500 μm-thick slices using a vibratome (Leica VT1000E), placed onto a phosphor imaging screen in a cassette (Molecular Dynamics, CA) and curated at room temperature for 48 h. Afterwards, the plates were analyzed employing a Phosphorimager (Amersham Bio-Science, NJ).

### Gene expression assay

Brain tissue was collected and added to a sterilized 5 mL cryotube. The brain sample was immediately stabilized in RNA-protect tissue reagent (Qiagen) on ice. After removing the RNA protective reagent, mRNA was extracted using an RNeasy Midi kit (Qiagen) via the manufacturer's protocol. During this process, genomic DNA was removed by DNase. The concentrations of extracted mRNA from all mouse brains were diluted to be identical. cDNA was prepared with SuperScript IV VILO Master mix (ThermoFisher Scientific) as in the manufacturer's protocol. TaqMan qPCR (Assay ID for PKM2: Hs00987261_g1) was performed to calculate the ΔCt of the target gene (PKM2) over the housekeeping gene (β-actin) and translocator protein (TSPO). For ssDNA quantification assays, the brain samples were immediately stabilized in Allprotect tissue reagen (Qiagen) at room temperature and ssDNA was extracted using a DNeasy Blood & Tissue kit (Qiagen) following the manufacturer's protocol. The concentrations of ssDNA from all mouse brains were diluted to be identical and TaqMan qPCR (Assay ID for PKM2: Hs00987261_g1) was performed to calculate the ΔCt of the target gene (PKM2) over the housekeeping gene (GCG).

### Fluorescence immunohistochemistry and confocal fluorescence microscopy

For transduction studies, either AAV9:CAG-mNG (5×10^11^ vg, n = 6) or AAV9:EF1A-PKM2 (5×10^11^ vg, n = 6) in saline (150 μL) were systemically injected into C57BL/6 mice via the tail vein. For fluorescence imaging of PKM2 protein, mice were euthanized after blood perfusion with 1xPBS at 3 weeks post AAV injection. Brains were collected and fixed in 4% paraformaldehyde (in PBS, pH 7) overnight and sliced to 100 μm-thick on a vibratome (Leica, VT-1000E). Sliced tissues were sequentially immersed in a blocking solution (5% donkey serum, 0.1% Saponin) in 1xPBS for 1 hour, a rabbit anti-PKM2 primary antibody (Cell Signaling Technology, 1:100, 1% donkey serum, 0.1% saponin in PBS) overnight in a fridge, in a goat anti-rabbit secondary antibody-Alexa488 conjugate (ThermoFisher Scientific, ab1500077, 1:250 1% donkey serum, 0.1% saponin in PBS) at room temperature for 2 hours, and in DAPI (1/5000, 1xPBS, 0.1% saponin) for 20 minutes. After each treatment, tissues were washed with 1xPBS three times. Stained tissues were mounted on glass slides with ProLong glass antifade mountant (ThermoFisher Scientific) and cured overnight at room temperature. Whole brain tissue images were acquired on a confocal microscope (Leica TCS SP8) operated by LAS X 3.5.5. software (Leica) with a 10x lens. For the fluorescent imaging of mNG protein expression, brain tissue was stained in DAPI (1/5000, 1xPBS, 0.1% saponin) for 20 minutes and then imaged following the above procedure.

### Western blot assays

Harvested brain tissue was immediately frozen under liquid nitrogen, transferred to ice, and N-PER neuronal protein extraction reagent containing Halt protease inhibitor (ThermoFisher Scientific) was added. After homogenization, the tissue was centrifuged at 10,000 g for 10 min at 4 °C and the supernatant was collected and characterized by a BCA assay to assess total protein concentration. Loading and reducing buffers were then added to the protein mixture following the manufacturer's protocol, and the protein samples were loaded into an SDS-PAGE gel (20 μg/well). After gel electrophoresis, the separated protein was transferred to a PVDF membrane (ThermoFisher Scientific) for Western blotting. PKM2 and β-actin were stained with a PKM2 monoclonal rabbit antibody (1:1000, Cell Signaling Technology) and rabbit anti-actin antibody (1:2000, Sigma Aldrich) as primary antibodies and goat anti-rabbit IgG (H+L) antibody HRP conjugate (1:2000, Thermo Fisher Scientific) as the secondary antibody. Protein bands were detected with a CCD detector (BioRad ChemiDoc XRS+) for further analysis after antibody and chemiluminescence substrate (BioRad) incubation.

### Statistics and reproducibility

All statistical analyses were performed with GraphPad Prism software (Prism 9.3). The statistical tests with confidence intervals, effect sizes, degree of freedom and P values can be found in the source data. Sample size for each experiment and the biological replicates across experiments are shown in the appropriate figure caption.

## Supplementary Material

Supplementary figures and list of materials and reagents.
