# Supplementary material for: PET imaging of focused-ultrasound enhanced delivery of AAVs into the murine brain
Source: Theranostics. 2023 Sep 25;13(15):5151–69. doi: 10.7150/thno.85549 (PMC10614693; doi:10.7150/thno.85549)
Supplement: Supplementary file 1 — Supplementary figures and list of materials and reagents. [file thnov13p5151s1.pdf]

Supplementary Information

PET imaging of focused-ultrasound enhanced delivery of AAVs into the murine brain

Javier Ajenjo[1*], Jai Woong Seo[1*], Josquin Foiret[1], Bo Wu[1], Marina Nura Raie[1], James Wang[1], Brett Zain Fite[1], Nisi Zhang[1], Rim Malek[1], Corinne Beinat[1], Noeen Malik[2], David Alexander Anders[2], Katherine W. Ferrara[1]

[1]Molecular Imaging Program at Stanford (MIPS), Department of Radiology, School of Medicine, Stanford University, Stanford, CA, USA.

[2]Stanford Cyclotron & Radiochemistry Facility (CRF), Department of Radiology, School of Medicine, Stanford University, Stanford, CA, USA.

* Authors contributed equally

Corresponding author: Katherine W. Ferrara
E-mail: kwferrara@stanford.edu

This PDF file includes:

- Supplementary Figures S1 to S9
- The list of key material and reagents

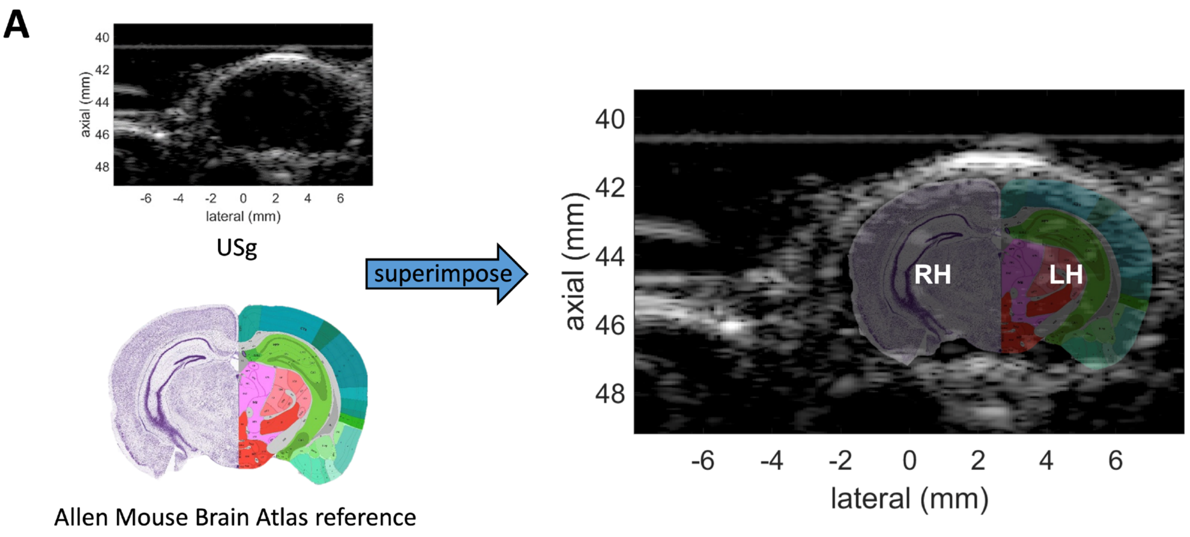

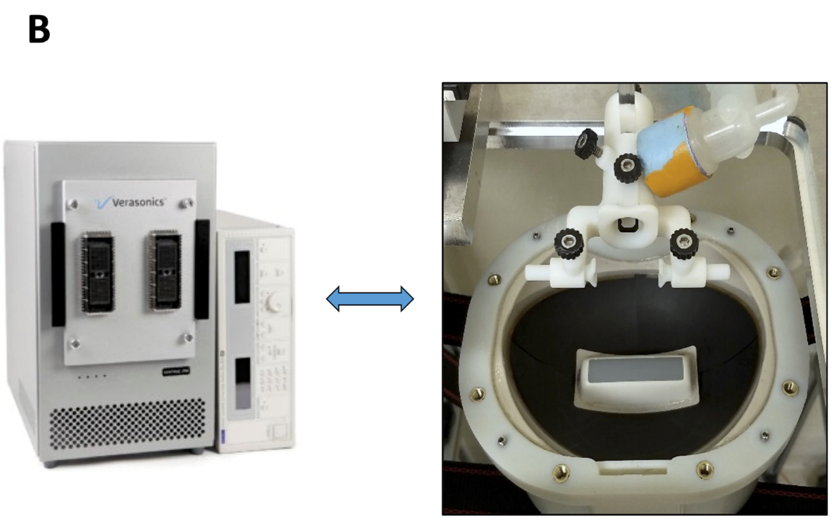

**Therapy array** (Imasonic, France) [1]	
Working frequency (MHz)	1.5
Number of elements	128
Radius of curvature (mm)	55
Aperture dimensions (mm)	105×85
-3 dB focus dimensions (mm)	0.4×0.7×2.7
Steerable volume (mm)	18×16×24

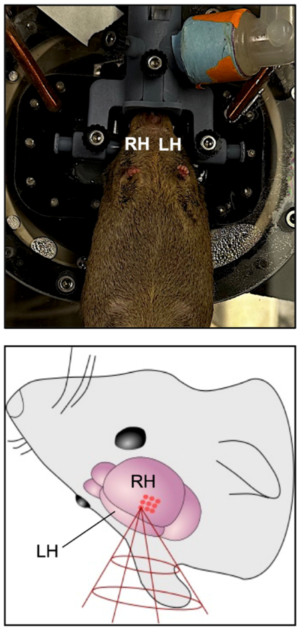

Figure S1. US-guided identification of FUS target areas: superimposition of US-guided images and anatomical annotations from the Allen Mouse Brain Atlas and Allen Reference Atlas – Mouse Brain (http://atlas.brain-map.org/, Mouse, P56, coronal) (**A**). Experimental therapy array setup for FUS treatment and array specifications (**B**). **Abbreviations:** RH: right hemisphere; LH: left hemisphere.

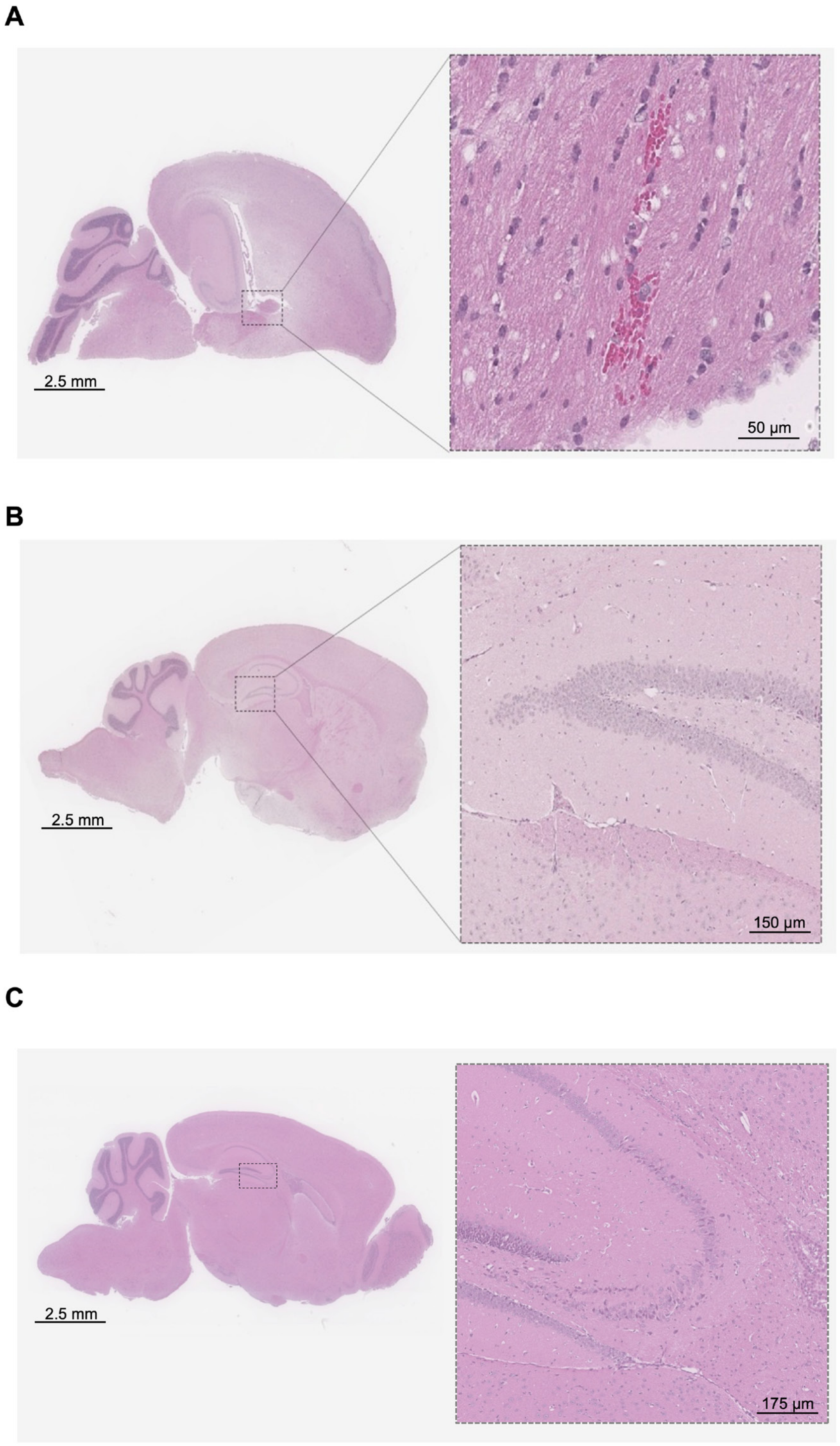

Figure S2. Hematoxylin and eosin-stained (H&E) sagittal brain slices of sonicated hemispheres at 740 (**A**), 600 (**B**), and 420 kPa (**C**). Augmented region of **A** shows red cell extravasation and apoptosis.

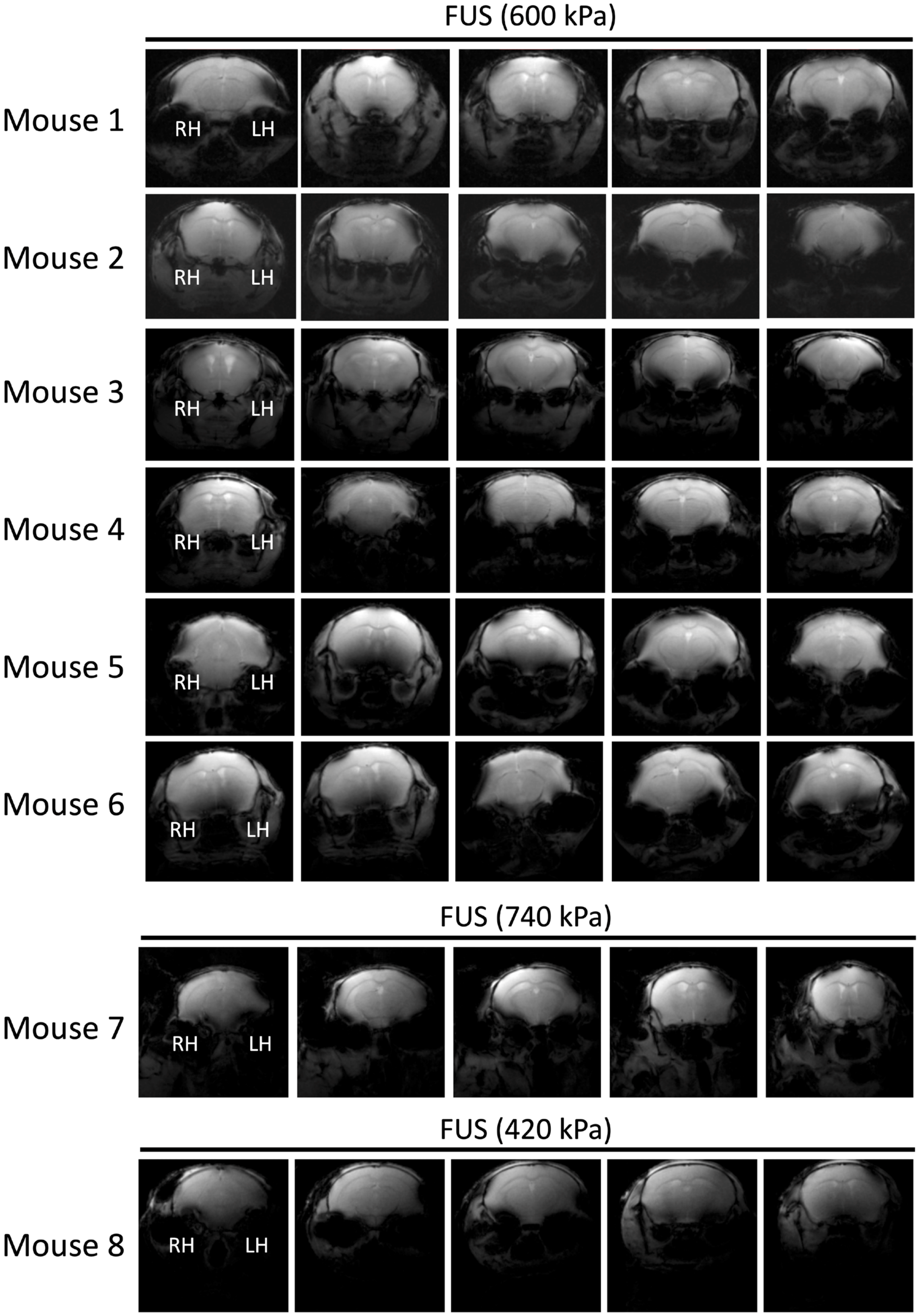

Figure S3. Representative planes of T2*-weighted MRI images post-FUS treatment at 600, 740 and 420 kPa, respectively, in the right hemisphere (RH) of treated subjects. For subjects treated at 420 and 600 kPa, changes in T2*-weighted MRI images due to insonation could not be detected at these FUS pressures.

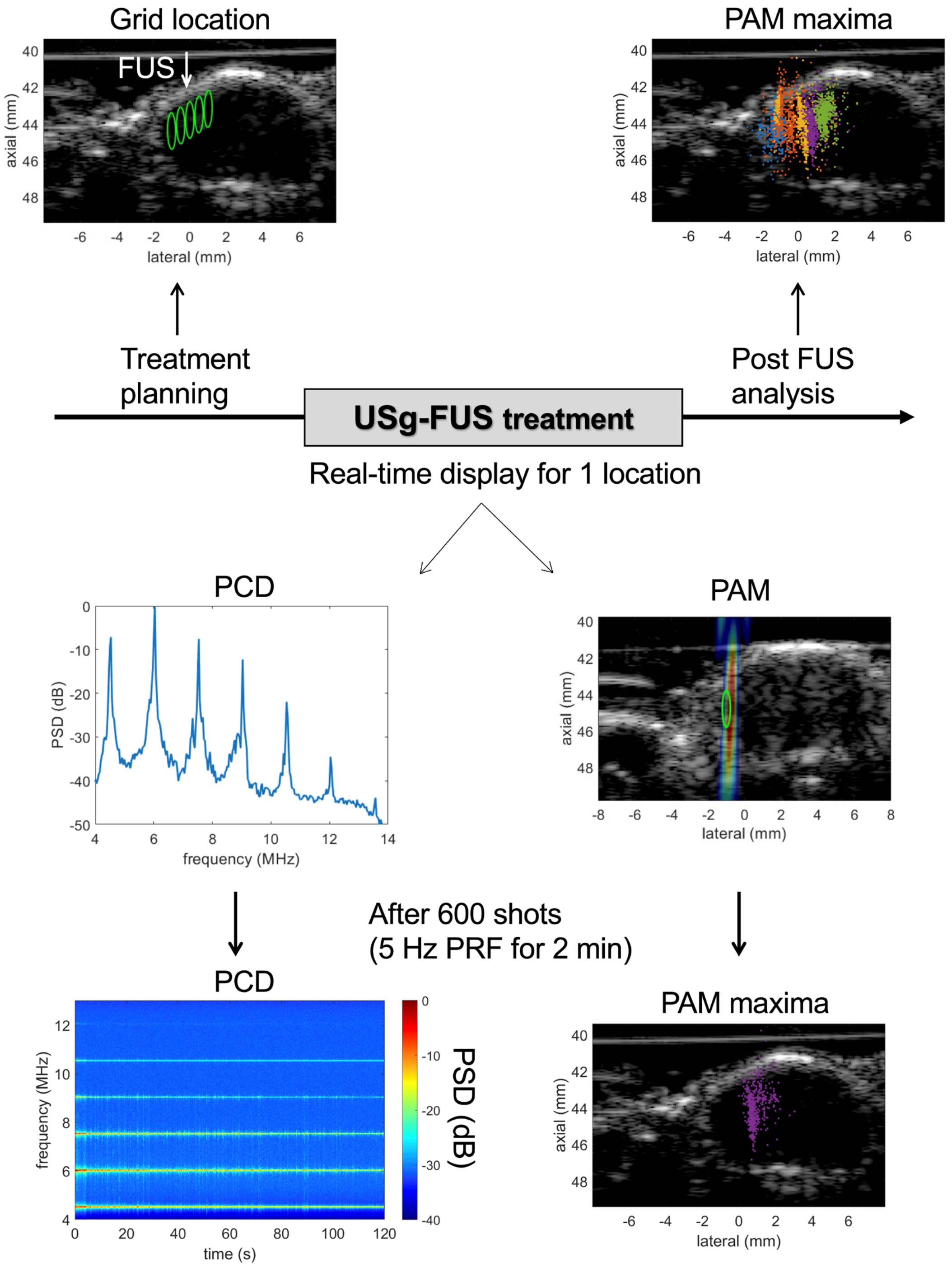

Figure S4. Methodology for the creation of the passive acoustic maps (PAMs) based on passive cavitation detection (PCD). This methodology is further detailed in Supplementary Figure S5. A treatment plan is created and RF data are collected with the imaging array while the therapeutic array is transmitting. The backscattered echoes primarily originate from oscillating microbubbles. Processing in the Fourier domain allows selection of specific frequency bands (like the harmonics here) and yields a passive acoustic map. This map is processed and overlaid on the ultrasound image in real-time during the treatment. Localizing the maximum of the passive acoustic map shows the agreement with the targeted area. We then create an integrated map with all grid positions.

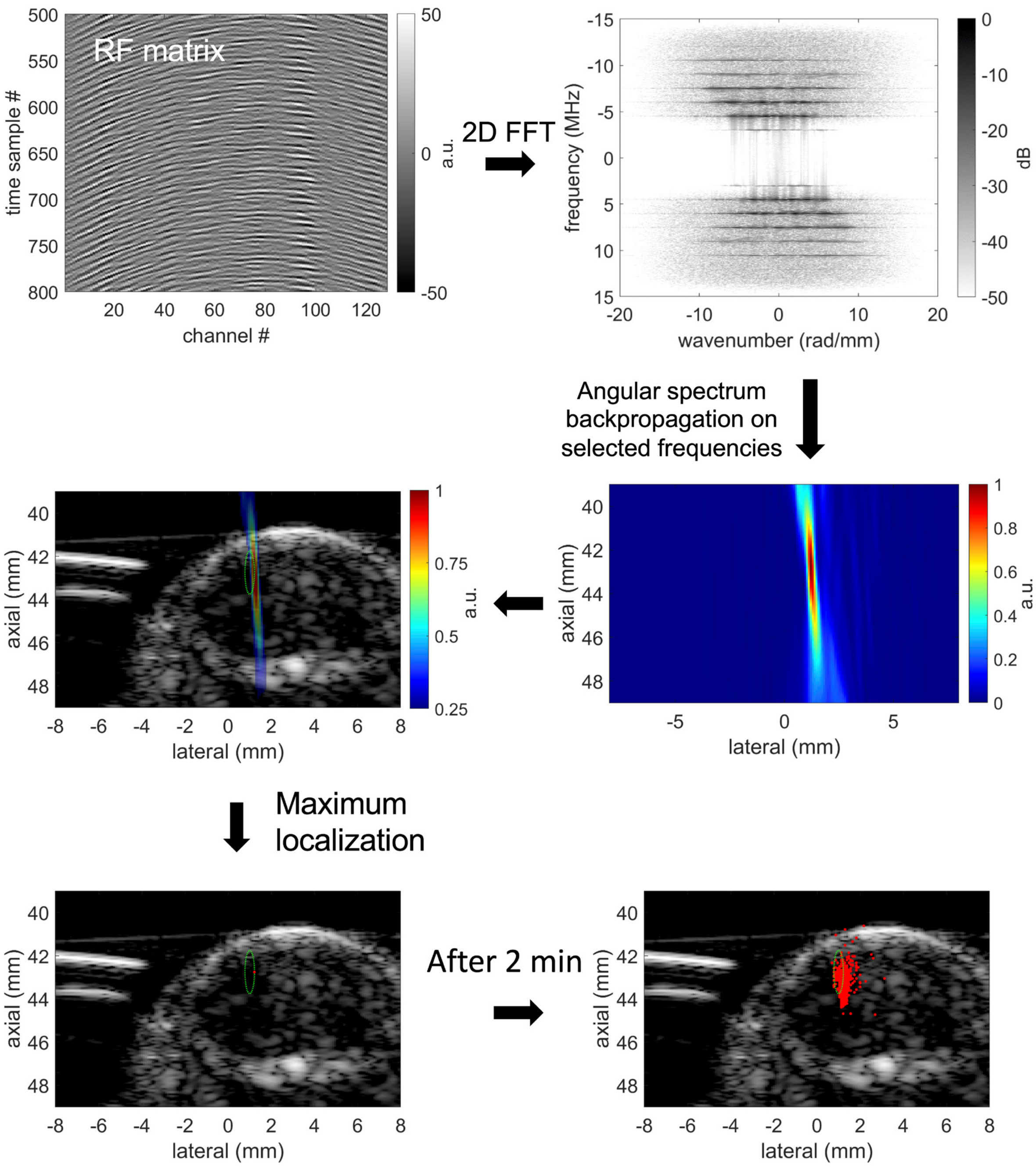

Figure S5. Steps for the angular spectrum passive acoustic mapping processing. First, RF data are collected with the imaging array while the therapeutic array is transmitting. The backscattered echoes primarily originate from oscillating microbubbles. The two-dimensional Fourier transform of the RF data is then utilized to perform the angular spectrum backpropagation. Processing in the Fourier domain allows selection of specific frequency bands (like the harmonics here) and yields a passive acoustic map. This map is processed and overlaid on the ultrasound image in real-time during the treatment. Localizing the maximum of the passive acoustic map shows the agreement with the targeted area.

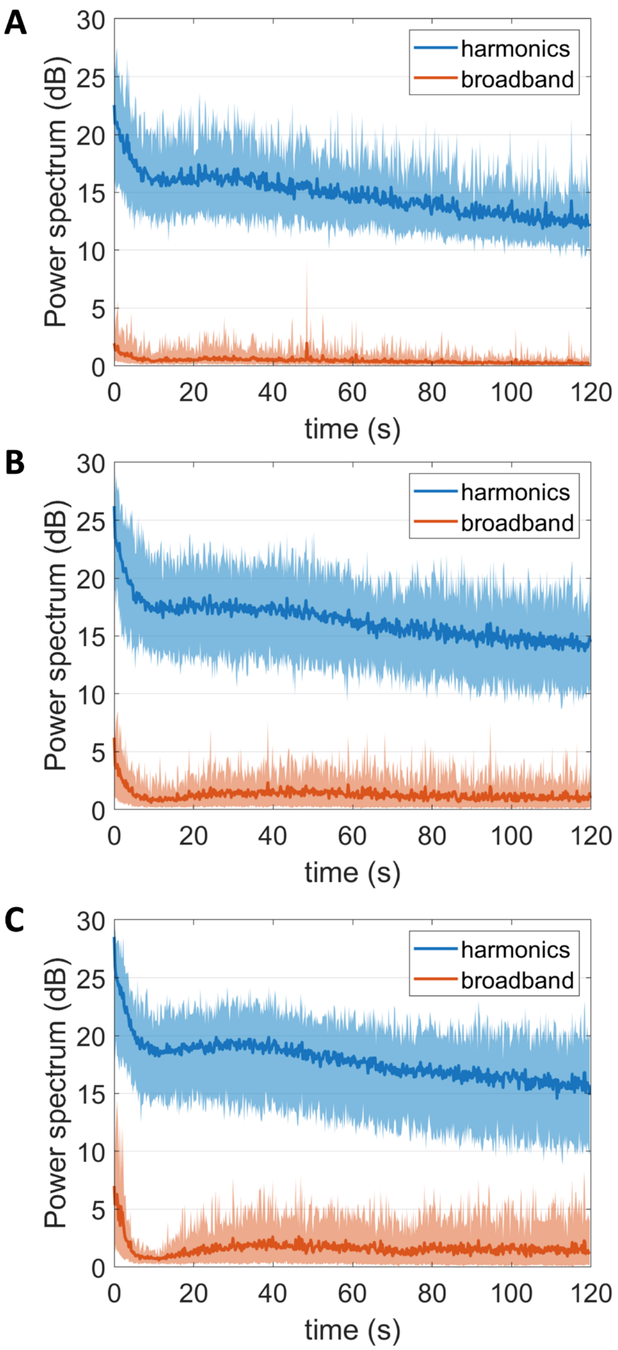

Figure S6. Comparison of average levels of acoustic emissions in the harmonics and the broadband frequency range for different acoustic pressures during FUS treatment over 2 minutes**.** The bold line represents the average value and the interval represent the range (minimum-maximum). Each pressure group pools 3 animals each having 5 focus locations: 420 kPa PNP (A), 600 kPa PNP (B), 740 kPa PNP (C).

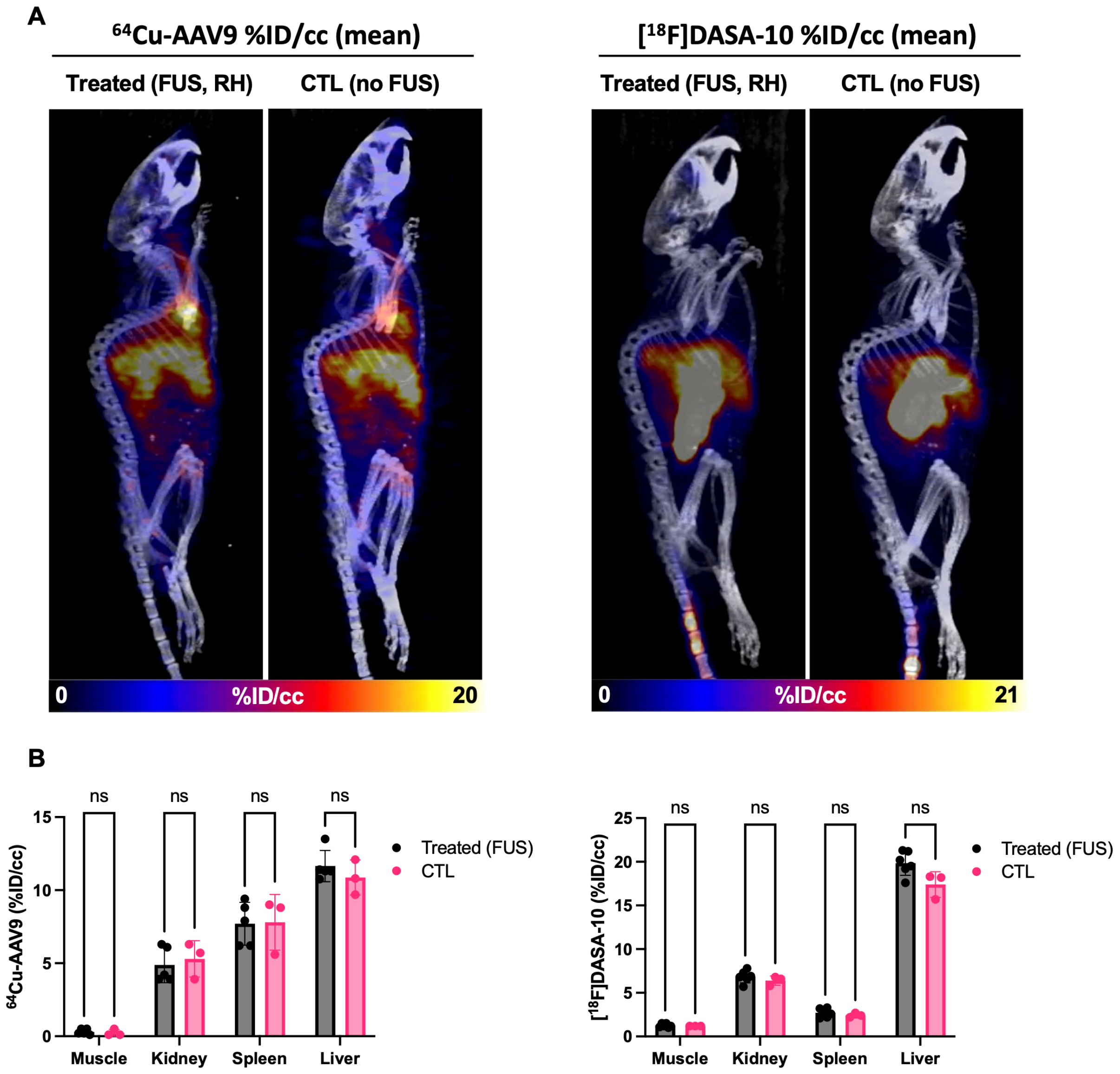

	^{64}Cu-AAV9 %ID/cc (mean)	
Organ	Treated (FUS, RH) (n = 5)	CTL (n = 3)
Muscle	0.3 ± 0.2	0.2 ± 0.2
Kidney	4.9 ± 1.2	5.3 ± 1.2
Spleen	7.7 ± 1.3	7.8 ± 1.9
Liver	11.7 ± 1.2	10.9 ± 1.2

	[^{18}F]DASA-10 %ID/cc (mean)	
Organ	Treated (FUS, RH) (n = 6)	CTL (n = 3)
Muscle	1.3 ± 0.2	1.2 ± 0.0
Kidney	6.9 ± 0.7	6.4 ± 0.5
Spleen	2.7 ± 0.5	2.4 ± 0.3
Liver	19.8 ± 1.4	17.4 ± 1.5

Figure S7. Off-target biodistribution (%ID/cc) of ^{64}Cu-AAV9 and [^{18}F]DASA-10 in C57BL/6 mice at 21h and 3-week post-injection, respectively. **A**. PET maximum intensity projection images (MIPs) of treated (FUS, RH) and untreated mice (CTL) for ^{64}Cu-AAV9 (left) and [^{18}F]DASA-10 (right) accumulation studies. **B**. Biodistribution (%ID/cc) in off-target organs for ^{64}Cu-AAV9 (left) and [^{18}F]DASA-10 (right) accumulation studies.

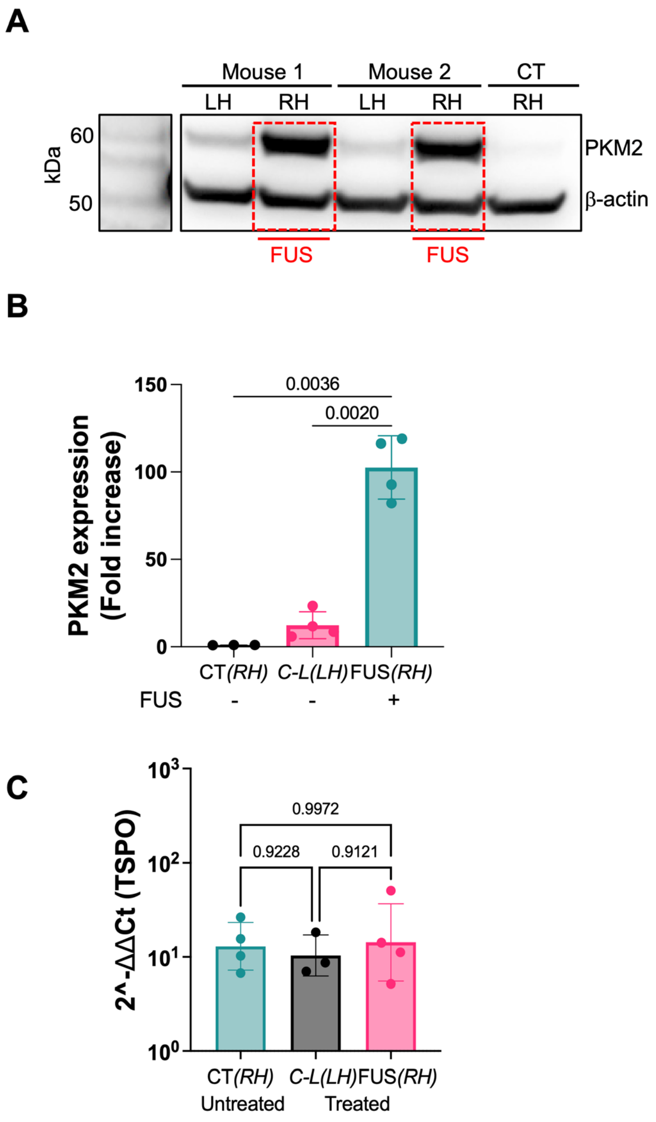

Figure S8. Protein and gene expression assessments from insonified and control untreated brain tissue. **A.** Protein expression assessment. Protein bands of PKM2 and β-actin control from mice brain at 3 weeks p.i. of AAV9. **B.** Reverse transcription quantitative polymerase chain reaction (RT-qPCR) assessment of expression: PKM2 mRNA fold increase in hemispheres of treated mice (n = 4) and no-FUS AAV9-injected control mice (n = 3). **C.** Gene expression of TSPO (translocator protein) from hemispheres of mice brain at 3 weeks p.i. pf AAV9. Brown-Forsythe and Welch ANOVA tests were performed for statistical analysis. ns: not significant (P> 0.05).

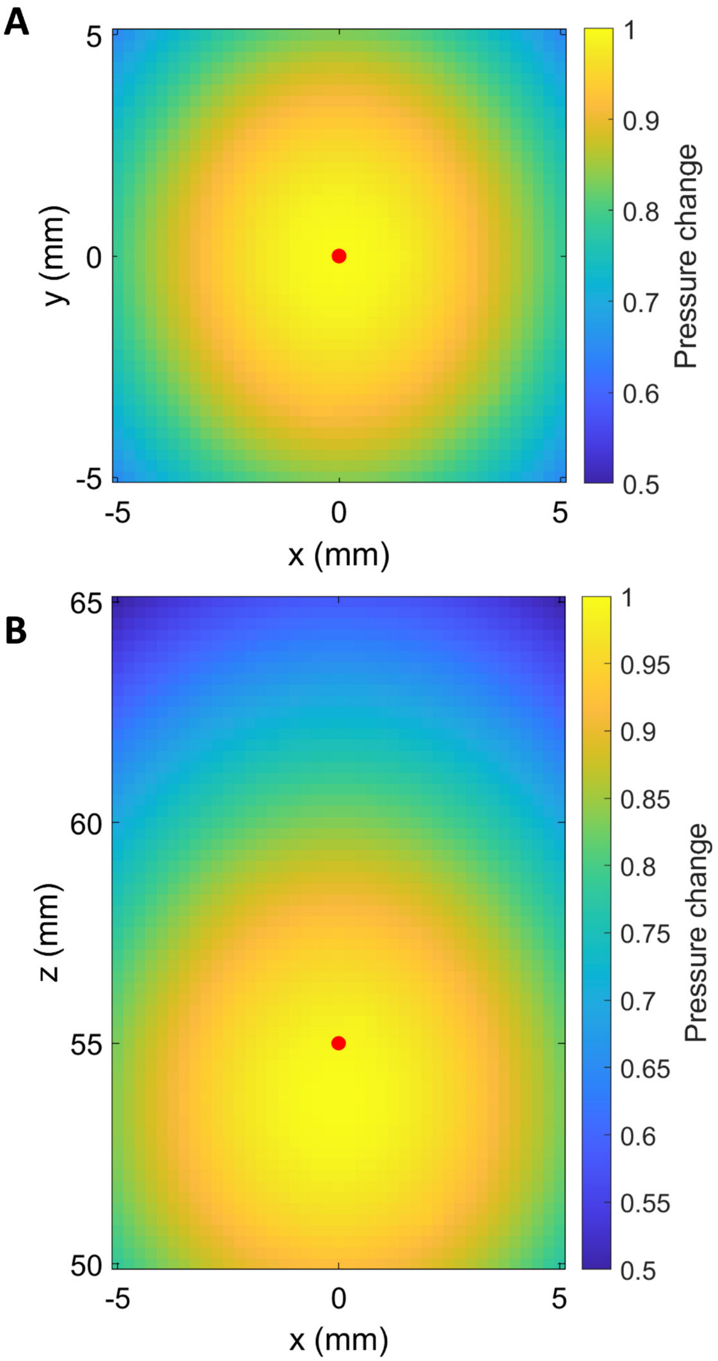

Figure S9. Evaluation of the acoustic pressure over space for calibration. Simulated acoustic pressure decreases when the beam is steered away from the array's geometric focus (indicated by the red dot). These simulations are integrated into the treatment planning to automatically compensate the driving signal to ensure a constant acoustic pressure for all grid points. Simulated pressure change in the x-y plane (lateral-elevational) (**A**) and the x-z plane (lateral-axial) (**B**), respectively.

List of materials and reagents

**Reagents**	**Source**	**Cat#**
Allprotect Tissue reagent	Qiagen	76405
DNase I recombinant for AAV titer	Sigma Aldrich	4716728001
RNase-free DNase set	Qiagen	79254
ProLong glass antifade mountant	Thermo Fisher scientific	P36980
Superscript IV VILO Master Mix	Thermo Fisher scientific	11756050
TaqMan™ fast advanced master mix	Thermo Fisher scientific	4444557
TaqMan™ primers and probe (WPRE)	Thermo Fisher scientific	Assay ID: APZTHAW
TaqMan™ primers and probe (PKM2)	Thermo Fisher scientific	Hs00987261_g1
TaqMan™ primers and probe (TSPO)	Thermo Fisher scientific	Mm00437828_m1
TaqMan™ primers and probe (b-actin)	Thermo Fisher scientific	Mm02619580_g1
RNAprotect Tissue reagent	Qiagen	76104
N-Per neuronal protein extraction reagent	Thermo Fisher scientific	87792
Blocking buffer	Thermo Fisher scientific	37542
Normal donkey serum	Jackson ImmunoResearch	017-000-121
1xDPBS without Calcium and Magnesium	Corning	20-031-CV
10xDPBS without Calcium and Magnesium	Cytiva	SH30378.03
10xTBST	Cell Signaling Technology	9997S
Bolt MES SDS running buffer	Thermo Fisher scientific	B0002
Bolt LDS sample buffer	Thermo Fisher scientific	B0007
Bolt antioxidant	Thermo Fisher scientific	BT0005
Bolt sample reducing agent	Thermo Fisher scientific	B0009
Saponin	Sigma-Aldrich	47036-50G-F
Pluronic F-68	Thermo Fisher scientific	24040032
Ammonium citrate	Fluka	09833
Anhydrous DMSO	Biotium	90082
Tetrazine-PEG5-NHS ester	Click Chemistry tools	1143-10
Clarity™ Western ECL Substrate	Bio-Red	1705060
DAPI	Invitrogen	D3571
4% PFA	Santa Cruz Biotechnology	
**Devices**	**Source**	**Cat#**
Mini-dialysis device (20k MWCO)	Thermo Fisher Scientific	88402
Amicon Ultra-15 Centrifugal filter unit 100kDa MWCO	EMD Millipore	UFC910024
Bolt 4-12% Bis-Tris plus gels	Thermo Fisher scientific	NW04120B
DNeasy Blood & Tissue kit	Qiagen	69504
RNeasy Midi Kit for mRNA extraction	Qiagen	75144
iBlot 2 Transfer stacks PVDF mini	Thermo Fisher Scientific	IB24002

**Instruments**	**Source**	**Cat#**
Vibratome	Leica	VT-1000E
PET/CT scanner	Siemens	-
Confocal microscope	Leica	TCS SP8
Gel imaging CDC detector	Bio-Rad	ChemiDoc XRS+
Real-time PCR detection system	Bio-Rad	CFX96 Touch
**Antibodies**	**Sources**	**Cat#; (RRID:AB)**
Goat anti-rabbit IgG (H+L) Alexa Fluor 488	Abcam	Ab150077
Pkm2 XP® Rabbit mAb	Cell Signaling Technology	4053S (D78A4)
β-Actin Rabbit mAb	Cell Signaling Technology	4970S (13E5)
Goat anti-rabbit IgG(H+L) secondary antibody HRP	Thermo Fisher Scientific	31460 (AB_228341)
